# Suppression of Vimentin Phosphorylation by the Avian Reovirus p17 through Inhibition of CDK1 and Plk1 Impacting the G2/M Phase of the Cell Cycle

**DOI:** 10.1371/journal.pone.0162356

**Published:** 2016-09-07

**Authors:** Hung-Chuan Chiu, Wei-Ru Huang, Tsai-Ling Liao, Hung-Yi Wu, Muhammad Munir, Wing-Ling Shih, Hung-Jen Liu

**Affiliations:** 1 Institute of Molecular Biology, National Chung Hsing University, Taichung 402, Taiwan; 2 Agricultural Biotechnology Center, National Chung Hsing University, Taichung 402, Taiwan; 3 Rong Hsing Research Center for Translational Medicine, National Chung Hsing University, Taichung 402, Taiwan; 4 Department of Medical Research, Taichung Veterans General Hospital, Taichung 402, Taiwan; 5 Department of Veterinary Medicine, National Pingtung University of Science and Technology, Pingtung 912, Taiwan; 6 The Pirbright Institute, Pirbright, United Kingdom; 7 Department of Biological Science and Technology, National Pingtung University of Science and Technology, Pingtung 912, Taiwan; Florida State University, UNITED STATES

## Abstract

The p17 protein of avian reovirus (ARV) causes cell cycle retardation in a variety of cell lines; however, the underlying mechanism(s) by which p17 regulates the cell cycle remains largely unknown. We demonstrate for the first time that p17 interacts with CDK1 and vimentin as revealed by reciprocal co-immunoprecipitation and GST pull-down assays. Both *in vitro* and *in vivo* studies indicated that direct interaction of p17 and CDK1/vimentin was mapped within the amino terminus (aa 1–60) of p17 and central region (aa 27–118) of CDK1/vimentin. Furthermore, p17 was found to occupy the Plk1-binding site within the vimentin, thereby blocking Plk1 recruitment to CDK1-induced vimentin phosphorylation at Ser 56. Interaction of p17 to CDK1 or vimentin interferes with CDK1-catalyzed phosphorylation of vimentin at Ser 56 and subsequently vimentin phosphorylation at Ser 82 by Plk1. Furthermore, we have identified upstream signaling pathways and cellular factor(s) targeted by p17 and found that p17 regulates inhibitory phosphorylation of CDK1 and blocks vimentin phosphorylation at Ser 56 and Ser 82. The p17-mediated inactivation of CDK1 is dependent on several mechanisms, which include direct interaction with CDK1, p17-mediated suppression of Plk1 by activating the Tpr/p53 and ATM/Chk1/PP2A pathways, and p17-mediated cdc25C degradation via an ubiquitin- proteasome pathway. Additionally, depletion of p53 with a shRNA as well as inhibition of ATM and vimentin by inhibitors diminished virus yield while Tpr and CDK1 knockdown increased virus yield. Taken together, results demonstrate that p17 suppresses both CDK1 and Plk1functions, disrupts vimentin phosphorylation, causes G2/M cell cycle arrest and thus benefits virus replication.

## Introduction

A well-characterized regulatory mechanism involves the phosphorylation and activation of phosphatase CDC25, which consequently dephosphorylates and activates CDK1 leading to cell entry into mitosis. During the normal cell cycle stages, cyclin B1 accumulates in the S and G2 phases to form an inactive mitosis-promoting factor (MPF) with CDK1 [1]. Dephosphorylation of CDK1at T14/Y15 sites eventually activates the CDK1/cyclin B1 complex [2]. Polo-like kinase 1 (Plk1) is a serine/threonine protein kinase and acts as an important regulator of several events during mitosis, especially in regulating mitotic entry and exit. Activation of the anaphase-promoting complex (APC) by Plk1 initiates anaphase and exit from mitosis [3]. To initiate mitosis, Plk1 is essential for CDC25C phosphorylation and mitotic cyclin at the G2/M boundary [4]. It has been suggested that depletion of Plk1 diminished vimentin-Ser 82 phosphorylation in mitosis, indicating that Plk1 regulates the mitotic elevation of vimentin-Ser82 phosphorylation [5]. The major antagonist of CDK1-cyclin B1 activity in mitosis is protein phosphatase 2A (PP2A) with a B55 regulatory subunit. A minimum PP2A-B55 activity is required at the mitotic entry to allow the phosphorylation of CDK1 substrates. It was discovered that inhibition of PP2A-B55 at mitotic entry is controlled by Greatwall kinase [6]. Plk1 is also involved in the restart of the cell cycle after DNA replication [7].

Nucleoporin Tpr is a 267 kDa protein that is a component of the nuclear pore complex (NPC) which localizes at intranuclear filaments or nuclear baskets [8]. This protein has been suggested to play a role in regulating nucleocytoplasmic transport of p53 [9]. More recent reports have suggested that Tpr depletion induces nuclear accumulation of p53 [9, 10]. The p53 is a well-known tumor suppressor protein and induces DNA damage in response to a variety of cellular stresses. Cumulatively, these activities result in the occurrence of apoptosis or cell cycle arrest at the G1/S and G2/M boundaries. In addition, p53 represses the expression of many genes required for cell survival and cell cycle progression. The report by McKenzie et al. demonstrated that p53 inhibits Plk1 gene expression by binding to its promoter region [11]. The Plk1 is a critical mediator of the G₂/M cell cycle transition that is inactivated and depleted as part of the DNA damage-induced G₂/M checkpoint. In normal conditions, Myt-1 would be phosphorylated by Plk1 and inactivated. Vimentin is the most abundant intermediate filament (IF) protein and is essential component of the cytoskeletal networks, together with actin filaments and microtubules [12]. The study by Yamaguchi et al. has advanced our further understanding of CDK1-induced vimentin-Ser 56 and Plk-induced vimentin-Ser 82 phosphorylation [5]. Plk1-induced phosphorylation of vimentin at Ser 82 is elevated from the metaphase of the cell cycle and maintained until the end of mitosis [1, 5].

Avian reovirus (ARV) contains 10 double-stranded RNA genome segments which are enclosed in a double protein capsid shell [13]. These ARV genome segments have been found to encode at least ten structural proteins and four nonstructural proteins. It was demonstrated by Huang and colleagues that ARVs enter host cells via the binding of σC to host receptor(s) and subsequently through caveolin 1-mediated endocytosis [14]. The S1 genome segment of ARV contains three open reading frames that are translated into p10, p17, and σC proteins, respectively [15]. The p17 protein is a 146-amino acid protein that continuously shuttles between the nucleus and the cytoplasm [16], making it available to participate in cellular processes such as DNA binding, gene transcription, and cell growth regulation. We have recently reported that p17 protein plays crucial roles in regulating the cell cycle, host protein translation, and autophagy [10, 17–19]. The p17 protein has been shown to cause cell cycle retardation in a variety of cell lines, partially via activation of the p53 pathway [17]. However, the precise mechanism(s) by which p17 regulates the cell cycle is not yet well understood. In this study, we have focused on the question of whether and how p17 regulates the G2/M phase of the cell cycle. Here, we report a novel function of p17 as a negative regulator of both CDK1 and Plk1. Molecular investigations revealed that p17 regulate the CDK1/Plk1-mediated inhibition of vimentin phosphorylation at Ser 56 and Ser 82 and thus results in the G2/M cell cycle arrest and benefits virus replication. Findings presented in this study have advanced our understanding on the p17-mediated suppression of both CDK1 and Plk1, and simultaneously activation of multiple signaling pathways. Moreover, p17 appeared to facilitate virus replication via induction of cell cycle arrest and cellular translation shutoff [10, 17, 18] and thus diverting the cellular machinery required for normal cell-cycling processes for virus replication.

## Materials and Methods

### Chemicals and Antibodies

Caffeine, an ATM kinase specific inhibitor, okadaic acid for inhibiting PP2A, 3,3’-iminodipropionitrile (IDPN) for vimentin disruption, and propidium iodide (PI) for cell staining were purchased from Sigma-Aldrich (St. Louis, USA). Proteasome inhibitor MG132 was from Calbiochem (San Diego, USA). Etoposide was used to stimulate DNA damage, while nocodazole was used to arrest cells in M phase. These chemicals were from EMD Biosciences, Inc. (San Diego, USA). The p17 monoclonal antibodies were produced previously by our laboratory. Rabbit anti-p-ATM (Ser 1981), rabbit anti-ATM, mouse anti-Chk1, mouse anti-p-Chk1, mouse anti-Chk2, mouse anti-p-Chk2, rabbit anti-CDC25C, mouse anti-CDK1, rabbit anti-p-vimentin (Ser 56), rabbit anti-p-vimentin (Ser 82), rabbit anti-vimentin, rabbit anti-p-p53 (Ser 15), rabbit anti-p53, rabbit anti-p-Plk1 (T210), rabbit anti-Plk1, rabbit anti-Myt1 were from Cell Signaling Technology (Danvers, USA). Rabbit anti-beta-actin antibody was from Millipore (Billerica, MA). Rabbit anti-p-CDC25C (Ser 216), rabbit anti-p-CDK1 (T14 and Y15), rabbit anti-nucleoporin Tpr, rabbit anti-p-p21 (T145), rabbit anti-p21 were from Santa Cruz Biotechnology (Santa Cruz, CA, USA). Rabbit anti-p-Myt1 (T495) antibody was from Sigma-Aldrich. Anti-mouse IgG (H+L) and anti-rabbit IgG (H+L) antibodies were purchased from Kirkegard & Perry Laboratories (Washington, DC., USA).

### Virus and Cell Lines

The S1133 strain of ARV was originally adopted in immortalized chicken embryo fibroblast (DF-1) cells. Both DF-1 and Vero cells were used and maintained in minimum essential medium supplemented with 10% fetal bovine serum (FBS), penicillin, and streptomycin at 37°C in a 5% CO_2_ humidified incubator. Cells were seeded 1 day before each experiment in 6-well plates with 5×10^5^ cells and grown at 37°C in a 5% CO_2_ humidified incubator.

### shRNA Constructs

To explore the role of Tpr, p53, and CDK1 in the regulation of mitosis and virus replication, Vero or DF-1 cells at 75% confluence were transfected with gene-specific shRNAs targeting Tpr, p53, CDK1, scrambled shRNA (29-mer non-effective scrambled pGFP-V-RS vector), and pGFP-V-RS vector, respectively. All the shRNAs and scrambled shRNAs (TR30013) were obtained from OriGene Co. (Rockville, USA) and constructed in the vector pGFP-V-RS (TR30007). Each shRNA kit containing four different shRNA sequences, targeting the respective genes, was tested in Vero cells. The one resulting in the most significant down regulation of respective protein expression was chosen and used in this study (Table 1).

**Table 1:** shRNAs used in this study.

Target gene	Cat. no	tube ID	Sequence (5’-3’)	Cell lines
Tpr	TG308677	GI334702	CTCAAGATTCCATTGGAGAAGGAGTTACC	Vero
Tpr	TG308677	GI334704	GGTGAAGATAGTAATGAAGGAACTGGTAG	DF-1
CDK1	TG320288	GI378373	AAACTACAGGTCAAGTGGTAGCCATGAAA	Vero
p53	TG320558	GI379451	CTCAGACTGACATTCTCCACTTCTTGTTC	Vero
p53	TG320558	GI379448	CAGCCAAGTCTGTGACTTGCACGTACTCC	DF-1

### Synthetic Peptide

To define the interaction domain of vimentin with p17, we performed *in vitro* binding assays using a synthetic peptide and purified GST-p17 fusion protein. The synthetic peptide vimentin (aa 45–65) (His_6_-RPSTSRSLYASSPGGVYATRS) was synthesized by Bio Basic Inc (Markham, Canada).

### Plasmid Construction and Co-immunoprecipitation Assays

The pcDNA3.1vector containing the p17 gene of ARV has been described previously [10, 19]. To study the region of p17 protein involved in the interaction with CDK1 and vimentin, a series of Flag-tagged p17 deleted fragments were constructed into pcDNA3.1 vector and confirmed by DNA sequencing. Experiments were initiated in serum-free medium for 2 hours followed by refreshing the medium containing 5% of FBS overnight once cell confluency reached around 75%. To investigate whether p17 interacts with CDK1 or vimentin and whether p17 affects CDK1-vimentin or Plk1-vimentin interactions, co-immunoprecipitation assays were carried out as described previously [10]. Immunoprecipitation was performed using the Catch and Release kit (Upstate Biotechnology) according to the manufacturer's protocol.

### Expression and Purification of Fusion Proteins

The p17 and p17-(1–60)-truncated gene of ARV as well as the cellular genes for CDK1, cyclin B1, vimentin, and vimentin-(1–90) genes were amplified from RNA extracted from Vero cells using PCR Primers are shown in Table 2. The amplified PCR products were cut with respective restriction enzymes and then introduced into the corresponding sites in either pET32a (Novagen, USA) or pGEX4T-1 (GE Healthcare Life Sciences, United Kingdom) vectors. Constructs were confirmed by DNA sequencing. The recombinant plasmid was then transformed into *E*. *coli* BL21(DE3). The transformed *E*. *coli* cells were grown in Luria-Bertani (LB) broth with 100 ug/ml of ampicillin at 37°C to an optical density of 0.6 and then induced with 0.4 mM of IPTG for 5 h at 28°C. To obtain soluble forms of GST-tagged p17, CDK1, cyclin B1, vimentin, and vimentin-(1–90) fusion proteins, cells were harvested by centrifugation followed by resuspension in lysis buffer (1x PBS, 0.2 mM PMSF, 1% Triton X-100, 0.5% Sodium lauroyl sarcosinate). After sonication, cell suspensions were centrifuged at 12,000 x g for 20 min at 4°C. Each supernatant was changed to 1x PBS with Amicon Ultra 0.5-ml 10k filters (Millipore) by adding the same volume of 1x PBS at least five times. The supernatant was applied to a glutathione-Sepharose 4B beads column (GE Healthcare Bio-Sciences). After washing beads with 1 x PBS washing buffer, the GST fusion proteins were eluted from the column with elution buffer (1x PBS, 10 mM reduced glutathione). In His-tagged p17 fusion proteins, cells were harvested by centrifugation, followed by resuspension in pET system lysis buffer (20 mM Tris-HCl pH 8.0, 300 mM NaCl, 0.2 mM PMSF, 10% glycerol, 5 mM imidazole) and sonicated. Cell suspensions were centrifuged at 12,000 x g for 20 minutes at 4°C. The supernatant was applied to a nickel column. After washing beads with 150 ml washing buffer, the TrxA-His-tagged p17 fusion protein was eluted from the affinity column with elution buffer (20 mM Tris-HCl pH 8.0, 300 mM NaCl, 0.2 mM PMSF, 10% glycerol, 200 mM imidazole). Finally, His-tagged p17 or truncated p17 fusion proteins as well as GST-tagged fusion proteins were changed to 1x PBS and filtered through Amicon Ultra 0.5-ml filters (Millipore). Samples were stored at -80°C freezer until further use.

**Table 2:** Primers used in co-immunoprecipitation for amplification of the respective targeted genes.

Gene	Sequence (5′-3′)*	Location	Expected size (bp)
p17^a^	F: CGGAATTCATGCAATGGCTCCGCCATACGA (EcoRI) R: GCTCTAGATCATAGATCGGCGTCAAATCGC (XbaI)	293–314 733–712	441
p17(1–118)^a^	F: CGGAATTCACAATGCAATGGCTCCGCCATACG (EcoRI) R: AAACTCGAGTCAGGATTGAGACCCGCCATCCCAATG (XhoI)	293–313 646–623	357
p17(27–146)^a^	F: CGGAATTCACAATGGCATCATTTACTGCTATAAC (EcoRI) R: AACTCGAGTCATAGATCGGCGTCAAATCGCC (XhoI)	371–390 733–711	363
p17(27–118)^a^	F: CGGAATTCACAATGGCATCATTTACTGCTATAAC (EcoRI) R: AAACTCGAGTCAGGATTGAGACCCGCCATCCCAATG (XhoI)	371–390 646–623	279
p17(1–60)^a^	F: CGGAATTCACAATGCAATGGCTCCGCCATACG (EcoRI) R: GCCTCGAGTCAAGATAACAGAGTAGG (XhoI)	293–313 473–459	183
p17(61–146) ^a^	F: CAGAATTCATGCAACCTTGTAG (EcoRI) R: AACTCGAGTCATAGATCGGCGTCAAATCGCC (XhoI)	476–486 733–711	261
p17^b^	F: CCCAAGCTTATGCAATGGCTCCGCCATACGA (HindIII) R: CCGCTCGAGTCATAGATCGGCGTCAAATCGC (XhoI)	293–314 733–712	441
p17(1–60)^b^	F: CGGAATTCACAATGCAATGGCTCCGCCATACG (EcoRI) R: GCCTCGAGTCAAGATAACAGAGTAGG (XhoI)	293–313 473–459	183
p17 ^c^	F: CCCGAATTCATGCAATGGCTCCGCCATACGA (EcoRI) R: CCGCTCGAGTCATAGATCGGCGTCAAATCGC (XhoI)	293–314 733–712	441
CDK1 ^c^	F: GGGGGATCCATGGAAGATTATACCAAAATAGAG (BamHI) R: GGCTCGAGCTACATCTTCTTAATCTGATTGTC (XhoI)	42–65 935–912	894
Cyclin B1 ^c^	F: GAAGAATCCATGGCGCTCCGAGTCACCAGGACC (BamHI) R: GGCTCGAGTTACACCTTTGCCACAGCCTTGGC (XhoI)	171–194 1472–1449	1302
Vimentin ^c^	F: CGGAATTCATGACCACCAGGTCCGTGTCCTCGTCC (EcoRI) R: GCGGCCGCTTATTCAAGGTCATCGTGATGCTGAG (NotI)	318–344 1712–1687	1395
vimentin (1–90) ^c^	F: CGGAATTCATGACCACCAGGTCCGTGTCCTCGTCC (EcoRI) R: AACTCGAGTCAGTCGGCCAGCGAGAAGTCCACCG (XhoI)	318–344 587–562	270

F: forward; R: reverse;

*:Uderlines indicate the restriction sites designed in the indicated primers

^a^: pcDNA3.1-flag (flag-tagged);

^b^: pET32a (His-tagged);

^c^: pGEX4T-1 (GST-tagged).

### GST Pull-down Assays

To perform *in vitro* GST pull-down assays using His-tagged p17, p17(1–60), GST-tagged CDK1, vimentin, and vimentin (1–90), 1 ug purified GST protein or GST fusion proteins (GST-CDK1, GST-vimentin, and GST- vimentin-1-90) were coupled to the glutathione-Sepharose 4B beads and incubated at 4°C overnight with 100 ng of purified TrxA-His-p17 or TrxA-His-p17-(1–60) fusion protein in binding buffer (20 mM Tris-HCl, 25 mM NaCl, 10% glycerol, 1 mM DTT, 1 mM EDTA, 1 mM PMSF, and 10 μg/ml cocktail protease inhibitor). The protein bound glutathione beads were then washed five times with binding buffer and eluted with elution buffer (50 mM Tris-HCl, pH 8.0, 10 mM reduced glutathione). Elution fractions were boiled and examined by Western blot assays.

### *In Vitro* Kinase Assays

To investigate the possibility that the interaction of p17 and CDK1 results in the inhibition of CDK1 kinase activity and vimentin phosphorylation at Ser 56, an *in vitro* kinase assay was performed using vimentin as a substrate [20]. GST-vimentin (2 μM) was incubated with purified GST-cyclin B1 (10 nM) and GST-CDK1 (10 nM) as well as purified TrxA-His-p17 or TrxA-His-p17-(1–60) proteins at 37°Cfor 30 min in cold kinase buffer (25 mM Hepes, pH 7.4, 25 mM β-glycerophosphate, pH 7.4, 25 mM MgCl_2,_ 0.1 mM Na_3_VO_4_, 0.5 mM DTT) that contained 40 μM ATP in a final volume of 25 μl. Both TrxA-His-p17-(1–60) and BSA were used as negative controls. The phosphorylated levels of vimentin were analyzed by Western blotting with an anti-vimentin (Ser 56) antibody.

### G0/G1 and G2/M Cell Synchronization

To assess the effect of ARV p17 protein on the regulation of cell cycle progression, DF-1 and Vero cells were G0/G1 phase synchronized using serum deprivation by maintenance of the cells in either DMEM containing no FBS (for Vero cells) or 5% FBS (for DF-1 cells) supplementation for 72 h. Cells were transfected for 18 h with constructs (p17 and vector only) after serum deprivation for 54 h. Cells were washed three times with PBS and fixed in PBS containing 70% ethanol at -20°C. Cells were then treated with 50 μg/ml RNase and stained with 1μg/ml propidium iodide for 30 min at 4°C in the dark. The samples were immediately analyzed using a flow cytometer (FACSCanto II, BD). The percentage of cells in each phase of the cell cycle was analyzed by BD FACSDiva^™^ Software. G2/M phase synchronization was performed during incubation of the cells in maintenance medium, which was supplemented with etoposide or nocodazole for 24 h. The percentage of cells in the G2/M phase of the cell cycle was analyzed.

### Cell Lysates Preparation and Western Blot Assays

Vero cells from one 6-well-dish were ARV-infected or p17-transfected for 24 hours and then collected and lysed in lysis buffer (50 mM Tris-HCl [pH 7.5], 150 mM NaCl, 1% Nonidet P-40, 0.5% sodium deoxycholate, and 0.1% sodium dodecyl sulfate (SDS) supplemented with complete protease inhibitor cocktail (Roche, Switzerland). Portions (30 μg) of total protein lysate from each treatment were quantified by a Bio-Rad protein assay (Bio-Rad Laboratories, USA), electrophoresed in 12% polyacrylamide gels at 70 V through the stacking gel and at 100 V through the resolving gels and then transferred to a PVDF membrane. Expression of each individual protein was examined using corresponding primary antibody and visualization by horseradish peroxidase (HRP) conjugated secondary antibody. After incubation with enhanced chemiluminescence (ECL plus) (Amersham/Pharmacia, Bucks, UK), the membrane was exposed to X-ray films (Kodak, Rochester, USA).

### Determination of Virus Titer

To determine the effect of Tpr, p53, and CDK1 on ARV replication, shRNAs were used to knockdown the respective targets in ARV-infected Vero cells. With the exception of CDK1, individual 24-well plates of cells were transfected with various shRNAs for 6 hours, followed by ARV infection at a multiplicity of infection (MOI) of 5 for 24 hours. In the case of CDK1, Vero cells were infected with ARV at a MOI of 5 for 3 hours, followed by CDK1 shRNA transfection for 18 hours. The effects of ATM inhibitor caffeine, vimentin inhibitor IDPN (1%), G2/M cell cycle inhibitors nocodazole (60 ng/ mL), and etoposide (2 μM) on ARV replication were also examined as described above. Vero cells were pretreated with the respective inhibitor for 1 hour, followed by ARV infection at a MOI of 5 for 24 hours. Virus titers were determined as described previously [10, 19].

### Statistical Analysis

Data from virus titer assays were evaluated for statistical significance using the Student's *t*-test. All of the values are expressed as the mean± SD of at least three independent experiments. The p values less than 0.05 were considered statistically significant.

## Results

### A Central region of p17 protein Is Required for CDK1 Binding

**Fig 1:**
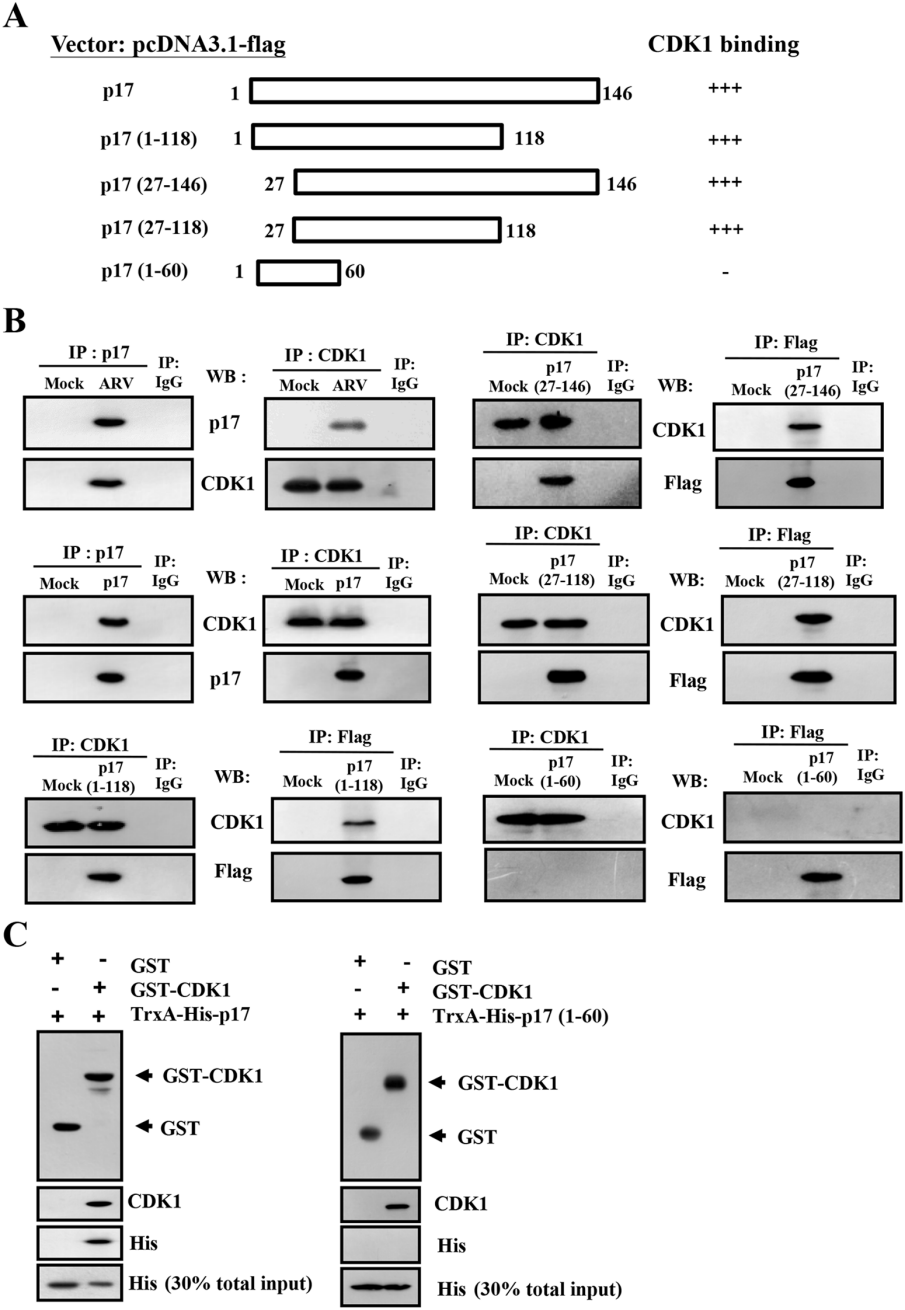
Identification of the binding region in p17 that is involved in CDK1 binding. (A) To define the region of p17 involved in CDK1 binding, a series of Flag-tagged p17 and truncated protein expression vectors were constructed. Schematic representation of p17 and deletion mutants is shown. The ability of p17 and truncated p17 mutants to interact with CDK1 is shown on the right hand side of the panel. +++: strong binding; -: no interaction (B) Three 6 cm dishes of Vero cells were either transfected with pcDNA3.1-p17 plasmid or infected with ARV at an MOI of 5 for 24 hours followed by co-immunoprecipitation with the respective antibodies. Cellular proteins co-immunoprecipitated by respective antibodies were analyzed by SDS-PAGE. Reciprocal co-immunoprecipitation assays in both ARV-infected and p17-transfected cells were performed with p17, Flag, and CDK1 antibodies, respectively. (C) *In vitro* GST pull-down assay as described in Material and Method section. The indicated proteins were examined by Western blot with the indicated antibodies. A total of 30% input of TrxA-His-17 or TrxA-His-17 (1–60) represented the internal loading control of input for p17 or 17 (1–60), respectively. Data shown are from three independent experiments. The uncropped blots with molecular weights are shown in S9 Fig.

We have previously reported that p17 retards cell cycle [17] and induces a down-regulation of CDK4 and cyclin D1 by activating the Tpr/p53 signaling pathway [10]. Therefore, we next propose that these actions of p17 are mediated through interaction of p17 to either CDK1 or other cell cycle-related proteins. As shown in reciprocal co-immunoprecipitation experiments, our results reveal that interactions between p17 and CDK1 occur in both ARV-infected and p17-transfected cells (Fig 1A and 1B). We subsequently sought to map the region of the p17 protein involved in CDK1 interactions using a series of Flag-tagged p17 deletion mutants. A schematic representation of p17 deletion mutants is shown in Fig 1A. The C-terminally truncated p17 mutant (1–118) and N-terminally truncated p17 mutants (27–146) were constructed and reported previously [10]. Deletion of the amino or carboxyl terminus of p17 in p17-(1–118), p17-(27–146), and p17-(27–118) did not interfere with the interactions of p17 and CDK1 whereas the p17-(1–60) deletion mutant was unable to exhibit CDK1 binding activity (Fig 1A and 1B). All together, these results suggest that the central region (aa 27–118) of p17 is necessary for interaction with CDK1. In an effort to further narrow down the interacting domain of p17, we demonstrated that amino or carboxyl terminus of the central region (aa 27–118) of p17 diminished its CDK1 binding activity (data not shown), implying that p17-CDK1 interaction is conformation-dependent. Since the reciprocal co-immunoprecipitation experiments above were unable to rule out the possibility that the interaction of p17 and CDK1 occurs indirectly due to the presence of other proteins, therefore, we carried out a GST pull-down assay. For this assay, constructs capable of expressing glutathione-S- transferase (GST)-CDK1, TrxA-His-p17, and TrxA-His-p17-(1–60) fusion proteins were generated. After cell pellets were treated with 0.5% sodium lauroyl sarcosinate, the soluble forms of expressed proteins were obtained. The integrity of the purified proteins was confirmed by SDS-PAGE and Coomassie brilliant blue staining (S1 Fig). A GST pull-down assay revealed that p17 was efficiently precipitated with GST-CDK1 (Fig 1C). Consistent with reciprocal co-immunoprecipitation results, a p17-(1–60) deletion mutant was not precipitated with GST-CDK1 (Fig 1C). GST alone did not bind to p17, indicating that the interaction was specific to p17 sequences.

### Amino Acids 1 to 60 of p17 Protein Are Required for Interaction with Vimentin-(45–65)

**Fig 2:**
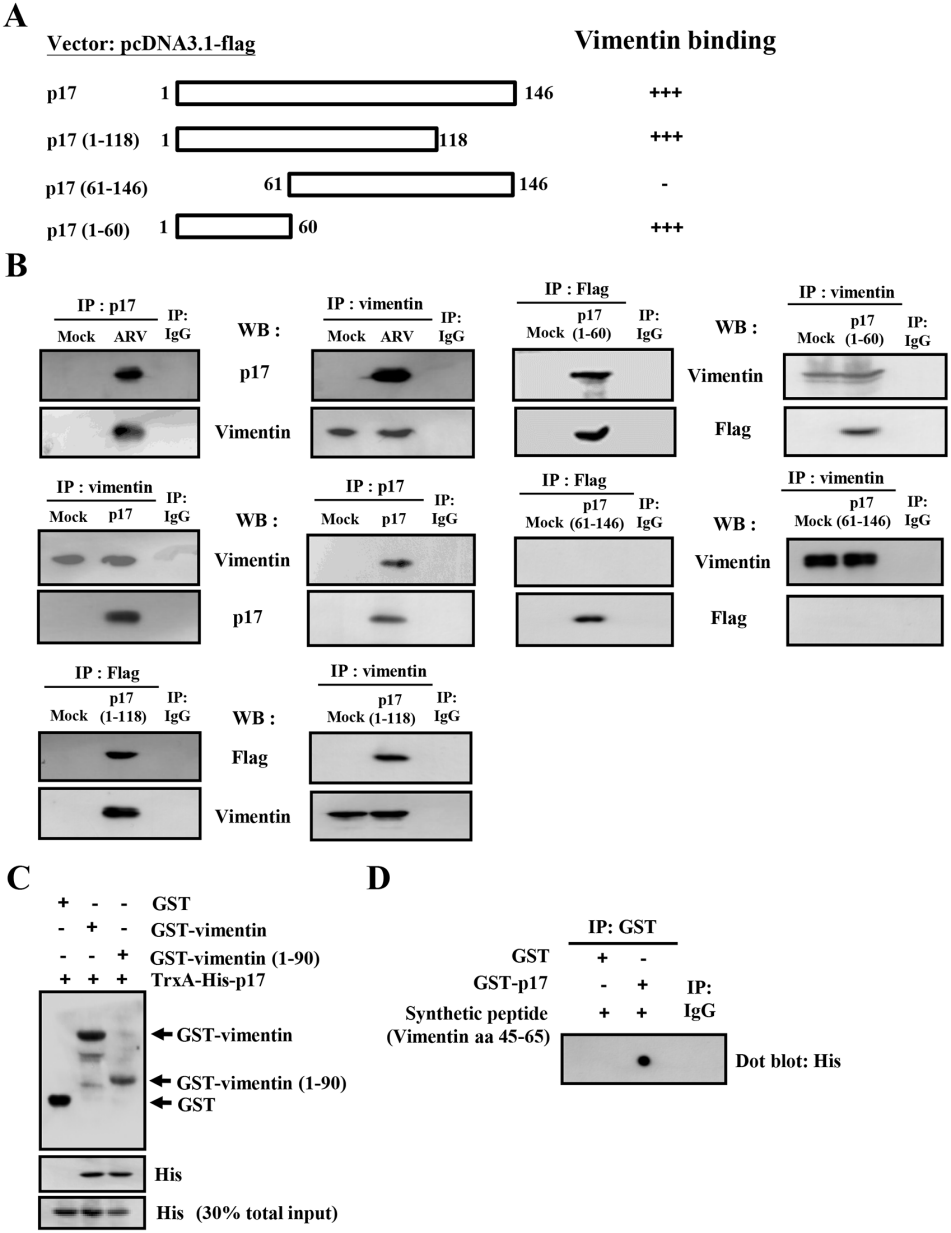
Identification of binding domains in p17 or vimentin involved in p17-vimentin interaction. (A) To map the region in p17 that is responsible for vimentin binding, a series of truncated versions of Flag-tagged p17 constructs were established. A schematic representation of p17 and p17 deletion mutants is shown. The ability of the p17 and truncated p17 mutants to interact with vimentin is shown on the right hand side of the panel. +++: strong binding; -: no interaction (B) Three 6 cm dish Vero cells were either transfected with pcDNA3.1-p17 and pcDNA3.1-p17 deletion plasmids or infected with ARV at a MOI of 5 for 24 hours and followed by co-immunoprecipitation assays with the indicated antibodies. Cellular proteins co-immunoprecipitated were analyzed by SDS-PAGE. Reciprocal co-immunoprecipitation assays in both ARV-infected and p17-transfected cells were performed with p17, Flag, and vimentin, respectively. (C) To define the binding region in vimentin, an i*n vitro* GST pull-down assay was carried out as described in the Material and Methods section. All indicated proteins were examined by Western blot assay. 30% of total input of TrxA-His-17 represented the internal loading control of input p17. (D) *In vitro* binding assays using a synthetic peptide vimentin-(45–65) and purified GST-p17 fusion protein were performed. The synthetic peptide vimentin-(45–65) and purified GST-p17 fusion protein were then subjected to analysis for their binding ability, as revealed by dot blot assay. The uncropped blots with molecular weights are shown in S9 Fig.

Our recent report demonstrated that p17 is able to co-immunoprecipitate with vimentin [10]. We subsequently wanted to map the regions of p17 protein involved in vimentin binding using a series of Flag-tagged p17 deletion vectors (Fig 2A). Cellular lysates from Vero cells infected with ARV or transfected with Flag-tagged p17 deletion vectors were reciprocally immunoprecipitated with anti-p17, anti-vimentin, and anti-Flag, respectively. Following immunoprecipitation with an anti-vimentin antibody, the full-length Flag-tagged p17 protein was detectable in both ARV-infected and p17-transfected cells. Deletion of the carboxyl terminus of p17 in p17-(1–118) and p17-(1–60) did not interfere with the interaction between p17 and vimentin while deletion of the amino terminus in p17-(61–146) abolished vimentin binding activity (Fig 2B). Taken together, our results indicate that the N-terminal region (1–60) in p17 protein is required for binding to vimentin. To further examine the vimentin domain involved in binding to p17, two plasmids capable of expressing glutathione-S-transferase (GST)—vimentin and GST-vimentin-(1–90) fusion proteins were constructed. Cell lysates were treated with 0.5% sodium lauroyl sarcosinate as described above. The GST-vimentin and GST-vimentin-(1–90) fusion proteins were purified using glutathione-Sepharose beads, followed by SDS-PAGE analysis and Coomassie brilliant blue staining to confirm integrity (S1 Fig). In this experiment, p17 was efficiently precipitated with GST-vimentin or GST-vimentin-(1–90) (Fig 2C). GST alone did not bind to p17, indicating that the interaction was specific to p17 sequences. To further define the domain of vimentin involved in p17 binding, synthetic peptide vimentin-(45–65) (His_6_-RPSTSRSLYASPGGVYATRS) and purified GST-p17 fusion proteins were used in dot blot assays. In this experiment, GST and IgG were included as negative controls. Dot blot assays revealed that vimentin-(45–65) displayed a strong interaction with p17 protein whereas GST alone did not exhibit p17 binding activity, suggesting that amino acids 45 to 65 of vimentin is required for direct interaction with p17 protein. Furthermore, immunofluorescence staining was performed to detect possible colocalization of p17 and vimentin. In Vero cells, vimentin shown diffused expression throughout the cytoplasm and p17 expressed in punctate in the perinuclear regions (S2 Fig). Nevertheless, immunofluorescence straining revealed that p17 and vimentin colocalized in Vero cells, further confirming the results demonstrated in the GST assays

### p17 Interacts Directly with CDK1 and Vimentin Which Leads to Inhibition of CDK1 Kinase Activity and Blocks Plk1 Recruitment to CDK1-Induced Vimentin Phosphorylation at Ser 56

**Fig 3:**
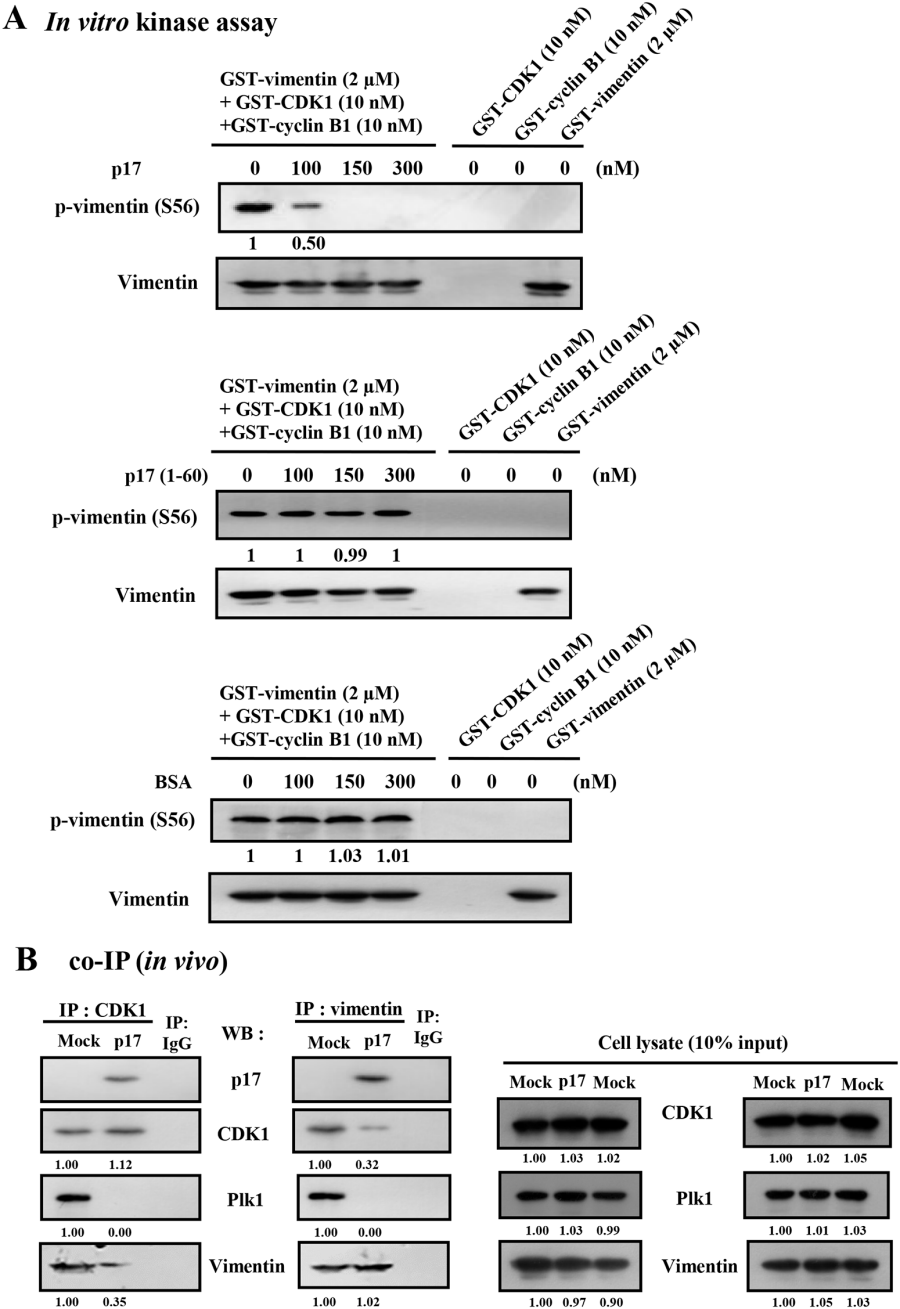
p17 inhibits CDK1 kinase activity which leads to inhibition of vimentin phosphorylation at Ser 56 and suppression of the association of CDK1-vimentin and Plk1-vimentin. (A) To explore whether p17 interacts with CDK1 leading to inhibition of CDK1 kinase activity and vimentin phosphorylation at Ser 56, an *in vitro* kinase assay using GST-vimentin as a substrate was performed. In the presence of TrxA-His-p17, the decrease in the level of vimentin-Ser56 phosphorylation was seen in a dose-dependent manner. The ki value for inhibition of CDK1/cyclin B1 by p17 that affects vimentin phosphorylation is about 100 nM. (B) Vero cells were transfected with pcDNA3.1-p17 for 24 hours followed by co-immunoprecipitation assays with the indicated antibodies. The immunoprecipitated proteins were separated by SDS-PAGE followed by Western blot assay with the respective antibodies. Rabbit IgG was used as a negative control. Reciprocal co-immunoprecipitation assays in p17-transfected cells were performed with CDK1 and vimentin, respectively. The representative data are from three independent experiments. The folds indicated below each lane were normalized against the values in the mock controls. The level of the indicated proteins in the mock control was considered 1-fold. The uncropped blots with molecular weights are shown in S10 Fig.

To explore whether the interaction of p17 with CDK1 and vimentin-(45–65) leads to inhibition of CDK1 kinase activity and abrogates vimentin phosphorylation at Ser 56, an *in vitro* kinase assay using vimentin as a substrate was performed. In this work, soluble forms of most expressed proteins were obtained by using 0.5% sodium lauroyl sarcosinate to treat protein samples. The integrity of the purified proteins was confirmed by SDS-PAGE and Coomassie brilliant blue staining (S1 Fig). With increasing concentration of p17, a decreased level of vimentin-Ser 56 phosphorylation was seen in a dose-dependent manner. The Ki value for inhibition of CDK1/cyclin B1 by p17 that affects the vimentin phosphorylation was estimated to be 100 nM (Fig 3A). As a negative control, BSA did not inhibit CDK1 kinase activity (Fig 3A). Consistent with results of reciprocal co-immunoprecipitation assays and GST pull-down assays (Fig 1B and 1C), p17-(1–60) deletion protein failed to inhibit CDK1 kinase activity. In addition, we found that CDK1/cyclin B1 complex kinase activity was also inhibited in the presence of p17 (S3 Fig).

**Fig 4:**
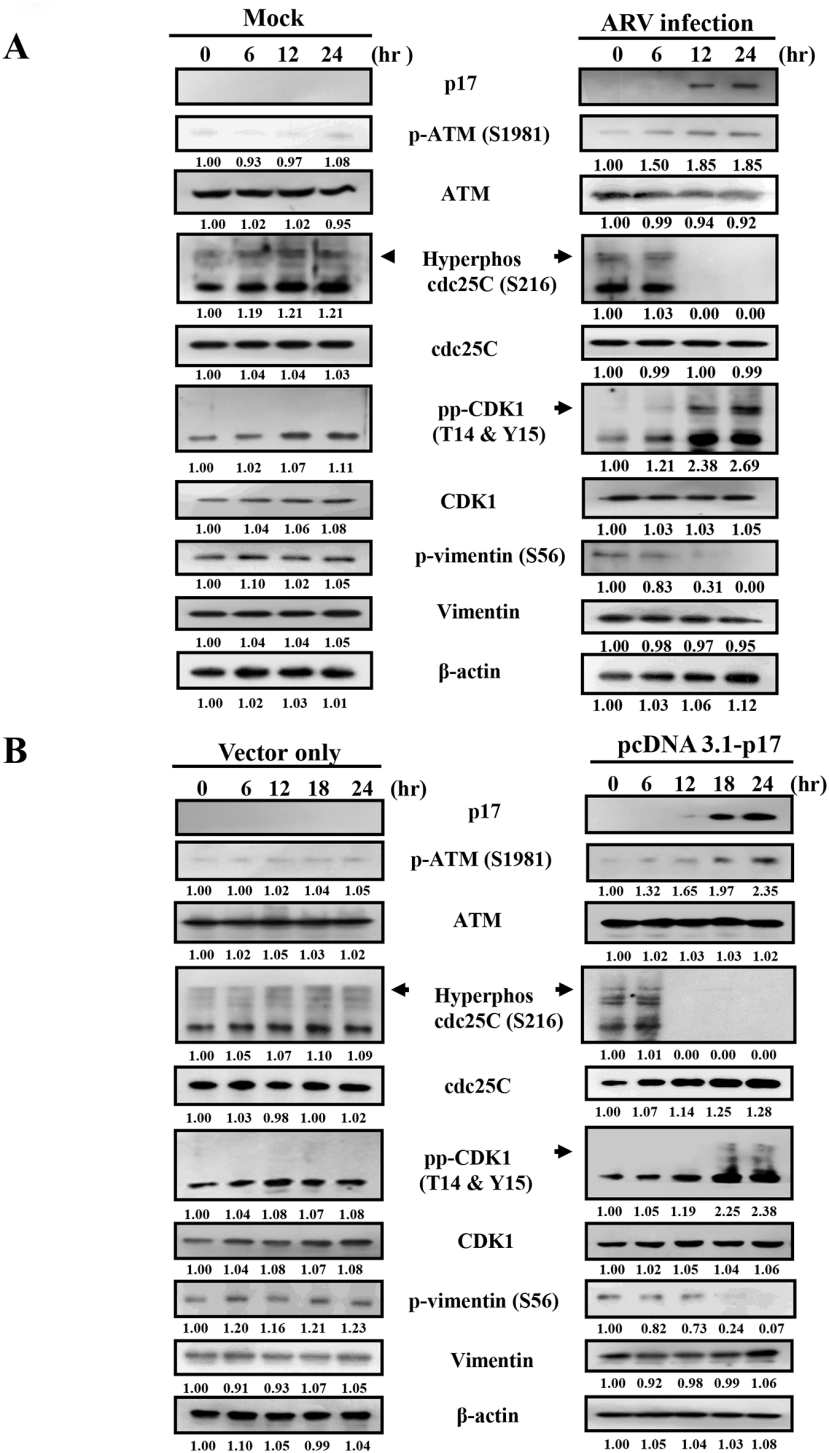
Inhibition of CDK1 by p17 is through inactivation of CDC25C. (A) The levels of p-ATM and cell cycle regulatory proteins CDC25C, CDK1, and vimentin-Ser 56 in ARV-infected and p17-transfected Vero cells were examined. Vero cells were either infected with ARV at a MOI of 5 (A) or transfected with p17. (B) Cells were collected for Western blots assays at the indicated time points. The protein levels were normalized to that for β-actin. The fold activation and inactivation indicated below each lane were normalized against the values at 0 h. The levels of the indicated proteins at 0 h were considered 1-fold. The uncropped blots with molecular weights are shown in S10 Fig.

Yamaguchi and colleagues have previously suggested that CDK1 controls mitotic vimentin phosphorylation not only by a direct enzyme-substrate reaction but also through Plk1 recruitment to phosphor-Ser-55 on vimentin via its polo box domain (PBD) [5]. Plk1 uses a PBD to recognize phosphorylated vimentin by CDK1, providing a functional and spatial coupling between Plk1 and CDK1 activity. Thus, we next wanted to examine whether p17 inhibits CDK1 kinase activity and binds to vimentin-(45–65), leading to blocking Plk1-vimentin interactions. Cellular lysates from Vero cells transfected with Flag-tagged p17 vector were reciprocally co-immunoprecipitated with anti-CDK1and anti-vimentin antibodies, respectively. Following immunoprecipitation with the anti-CDK1 antibody, p17 was efficiently precipitated with cellular CDK1 in p17-transfected cells (Fig 3B, left panel) while only a low level of vimentin was detected by Western blot assays compared to that in mock-transfected cells (Fig 3B, right panel). Reciprocally, p17 could also be pulled down in p17-transfected cells when an antibody against vimentin was used for immunoprecipitation (Fig 3B, right panel). p17 was detected as a protein associated with vimentin. Only a low level of CDK1 was detected compared to that in mock-transfected cells (Fig 3B, right panel). Our results reveal that Plk1 was neither precipitated with CDK1 nor vimentin in p17-transfected cells. Since total levels of CDK1 or vimentin in infected or transfected cells were unchanged (Fig 4A and 4B), we conclude that p17 weakens the interaction of CDK1-vimentin, thereby reducing vimentin phosphorylation at ser56. Our results suggest that p17 may occupy the binding site of Plk1 in vimentin or may reduce expression levels of Plk1, thereby blocking or reducing the binding of PBD of Plk1 to vimentin. Taking all findings together, we conclude that p17 binds to CDK1and vimentin (aa 45–65), both of which inhibit the CDK1-vimentin enzyme-substrate reaction, leading to blockade of Plk1 recruitment to CDK1-induced vimentin phosphorylation.

### ARV Infection and p17 Transfection Reduce Phosphorylation of Vimentin at Ser 56 by Upregulating the ATM/chk1/cdc25C Signaling Pathway

In addition to direct inhibition of CDK1 kinase activity, we hypothesized that p17 reduces CDK1 activity due to other factors such as protein degradation or kinase inactivation by inhibitory hyperphosphorylation. We therefore analyzed the upstream signaling pathways and upstream kinase/phosphatase networks. The levels of phosphorylated ATM, Chk1/2, CDC25C, CDK1 and vimentin revealed by immunoblot assay at indicated time points were examined. In comparison to mock-infected or mock-transfected control cells, we observed increased phosphorylation of ATM, Chk1/2, and CDK1 and decreased phosphorylation of vimentin (Ser 56) and CDC25C and total CDC25C in both ARV-infected and p17-transfected Vero cells 12 hours post infection or post transfection (Fig 4A, 4B and S4 Fig). Increased inhibitory phosphorylation of CDK1 following ARV infection and p17 transfection was detected, suggesting that in addition to direct inhibition of CDK1 kinase activity, p17 inhibits CDK1 by preventing CDK1 dephosphorylation via inactivation of phosphatase cdc25C

To date, a variety of checkpoint proteins have been identified as substrates for ATM and ATR kinases, including the checkpoint kinases Chk1/2, as well as H2AX and p53 [21, 22]. ATM phosphorylates Chk 2 at several sites including Thr 68 [7, 23, 24]. It was found that chk1 is phosphorylated at Ser 345 by ATR in response to UV light and hydroxyurea, leading to a 3-5-fold increase in Chk1 activity [22, 24]. In our previous study, we demonstrated that ARV S1133 increases the level of -Chk1 Ser 317/345 and Chk2 T68 phosphorylation in an ATM- dependent fashion [25]. Here, we further determine that p17 is the viral protein responsible for activation of ATM and chk1/2. The levels of p-ATM and p-Chk1/2 were diminished upon ATM inhibitor caffeine treatment in both ARV-infected and p17-transfected Vero cells (S4 Fig). Our data further confirm that ARV p17 plays an important role in upregulating the ATM/Chk1/2 signaling pathway.

**Fig 5:**
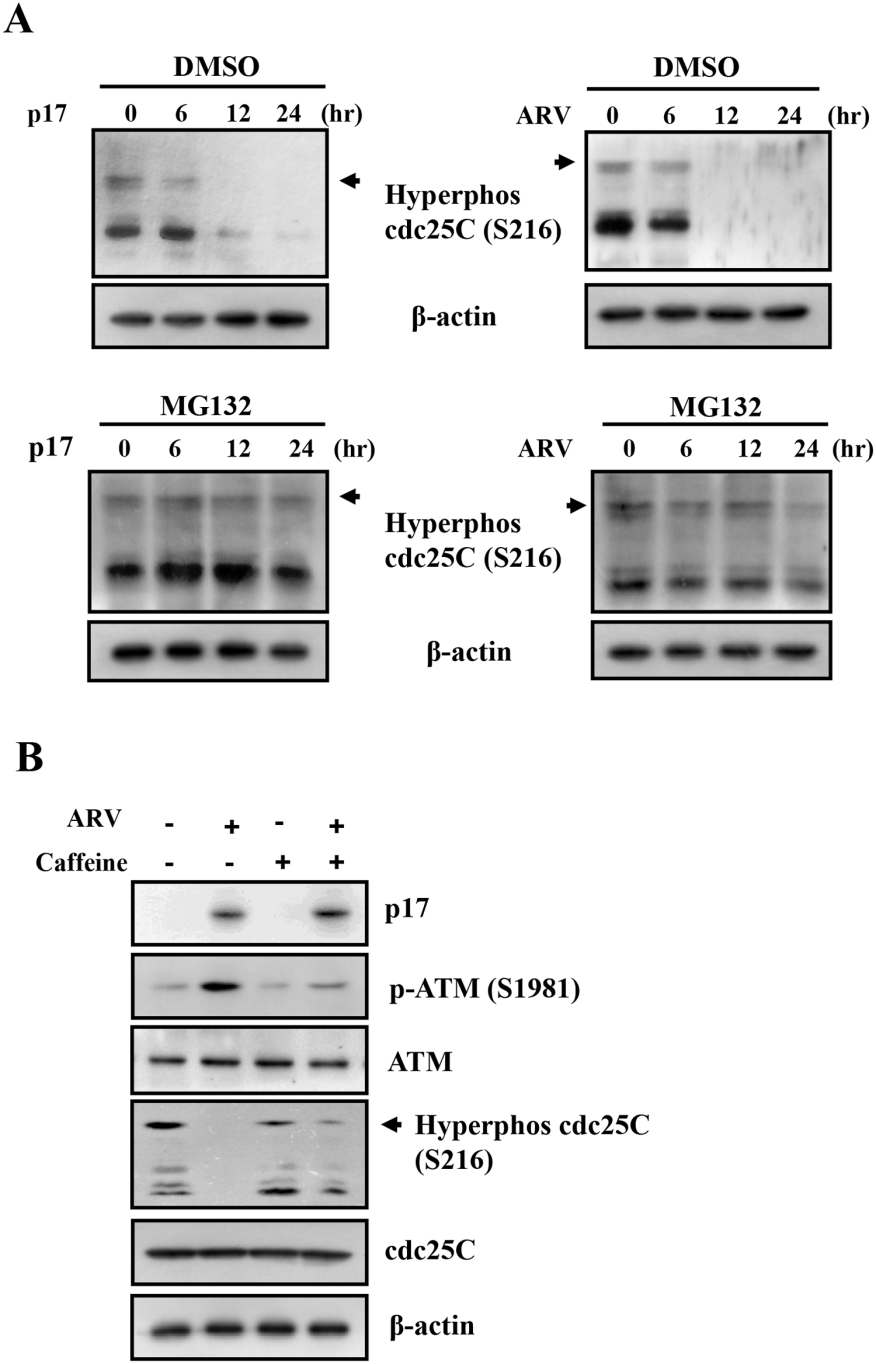
p17 mediates CDC25C degradation via the ubiquitin-proteasome pathway. (A) To study whether p17-mediatedCDC25C degradation via the ubiquitin-proteasome pathway, pretreatment with MG132 (25 uM) was performed in ARV-infected and p17-transfected vero cells. Total cell lysates were collected for analyzing the phosphorylated form of CDC25C. Experiments were repeated three times, and representative blots are shown. (B) Vero cells were pretreated with caffeine (2 mM) for 2 hours, and then infected with a MOI of 5 for 24 h. Total cell lysates were collected for analyzing the phosphorylation form of ATM and CDC25C. The experiments were repeated three times, and representative blots are shown. The uncropped blots with molecular weights are shown in S11 Fig.

Some other reports have suggested that ATM phosphorylates Chk, leading to phosphorylation of cdc25C at Ser 216 by chk1 and facilitating its binding to a 14-3-3 group of proteins that inactivate it through cytoplasmic sequestration [26–28]. Because an earlier study suggested that phosphorylation of CDC25C at Ser 216 is required for its translocation from the nucleus to the cytoplasm for degradation by the ubiquitin-dependent proteasome system [29], we further investigated whether p17 mediates CDC25C degradation through the ubiquitin-dependent proteasome system by using a proteasome inhibitor MG132 [30]. In the current study, we uncovered that MG132 could restore the phosphorylation of CDC25C at Ser 216 to prevent degradation of CDC25C in both ARV-infected and p17-transfected cells (Fig 5A, lower panels) compared to mock-treated controls (Fig 5A, upper panels). The results suggest that p17 facilitates CDC25C degradation via the ubiquitin-proteasome pathway. To confirm that CDC25C regulated by ATM is a dual-specificity protein phosphatase regulating entry into mitosis by dephosphorylating the protein kinase CDK1 [31], we further explored whether ATM is an upstream signal that regulates CDC25C. Thus, we used caffeine to inhibit ATM in ARV-infected Vero cells. The results shown in Fig 5B reveal that inhibition of ATM by caffeine restored partially the phosphorylated form and protein level of CDC25C. Taken together, our results demonstrate that ARV p17 inactivates CDK1 in part through inactivation of CDC25C by activating the ATM/Chk1 signaling pathway.

### Downregulation of Tpr and Upregulation of p53 by p17 Decreases Vimentin Phosphorylation at Ser 56

**Fig 6:**
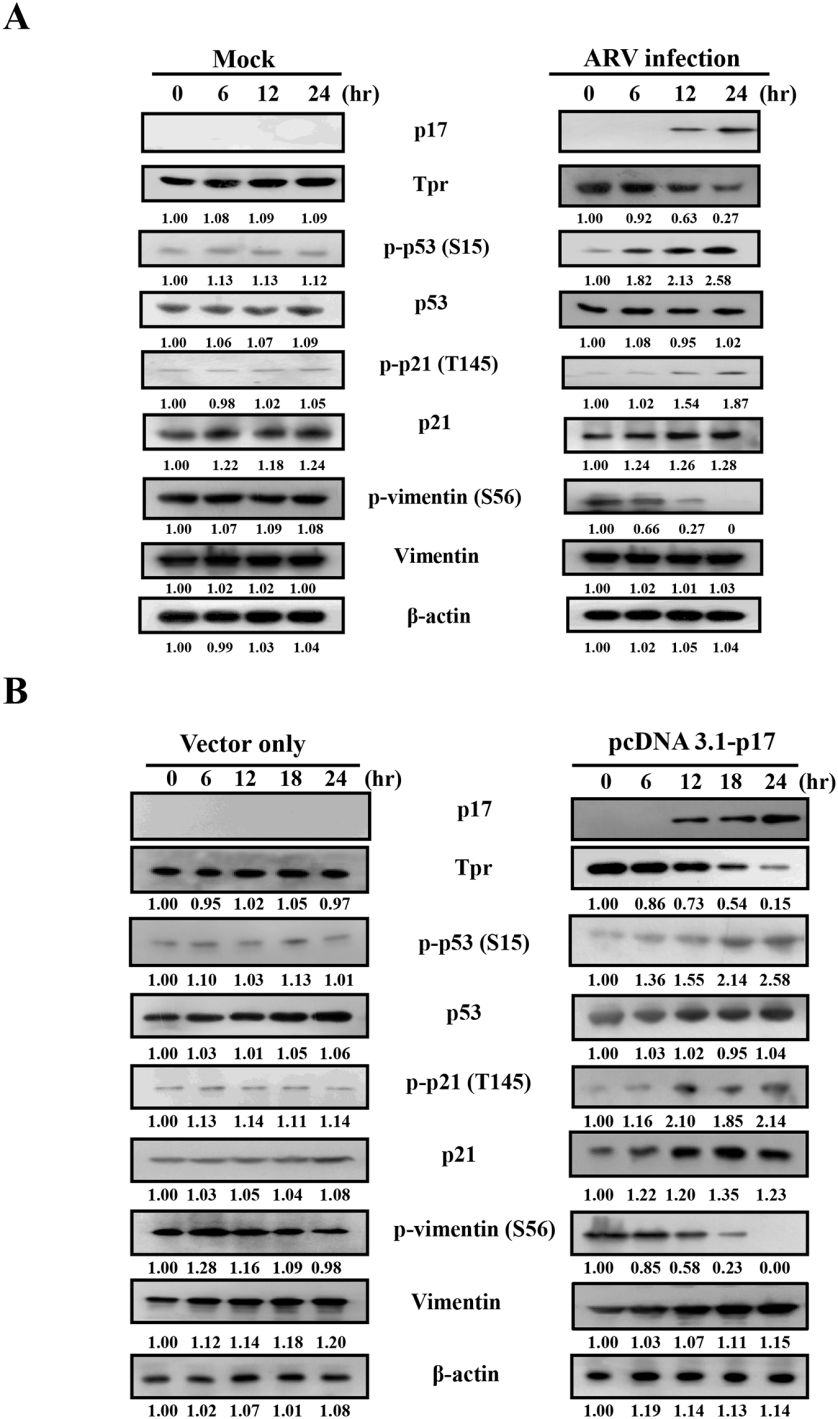
ARV infection and ARV p17 transfection inhibits nucleoporin Tpr and activation of p53 and p21. The levels of nucleoporin Tpr and the levels of p-p53, p-p21, and p-vimentin (Ser 56) in both ARV-infected (A) and p17-transfected Vero cells were examined. Vero cells were either mock-infected or ARV-infected with a MOI of 5 at the indicated time points. Phosphorylation and protein levels were analyzed by Western blot assays with the respective antibodies. The protein levels were normalized to that for β-actin. The fold activation and inactivation indicated below each lane were normalized against the values at 0 h. The levels of the indicated proteins at 0 h were considered 1-fold. The uncropped blots with molecular weights are shown in S11 Fig.

**Fig 7:**
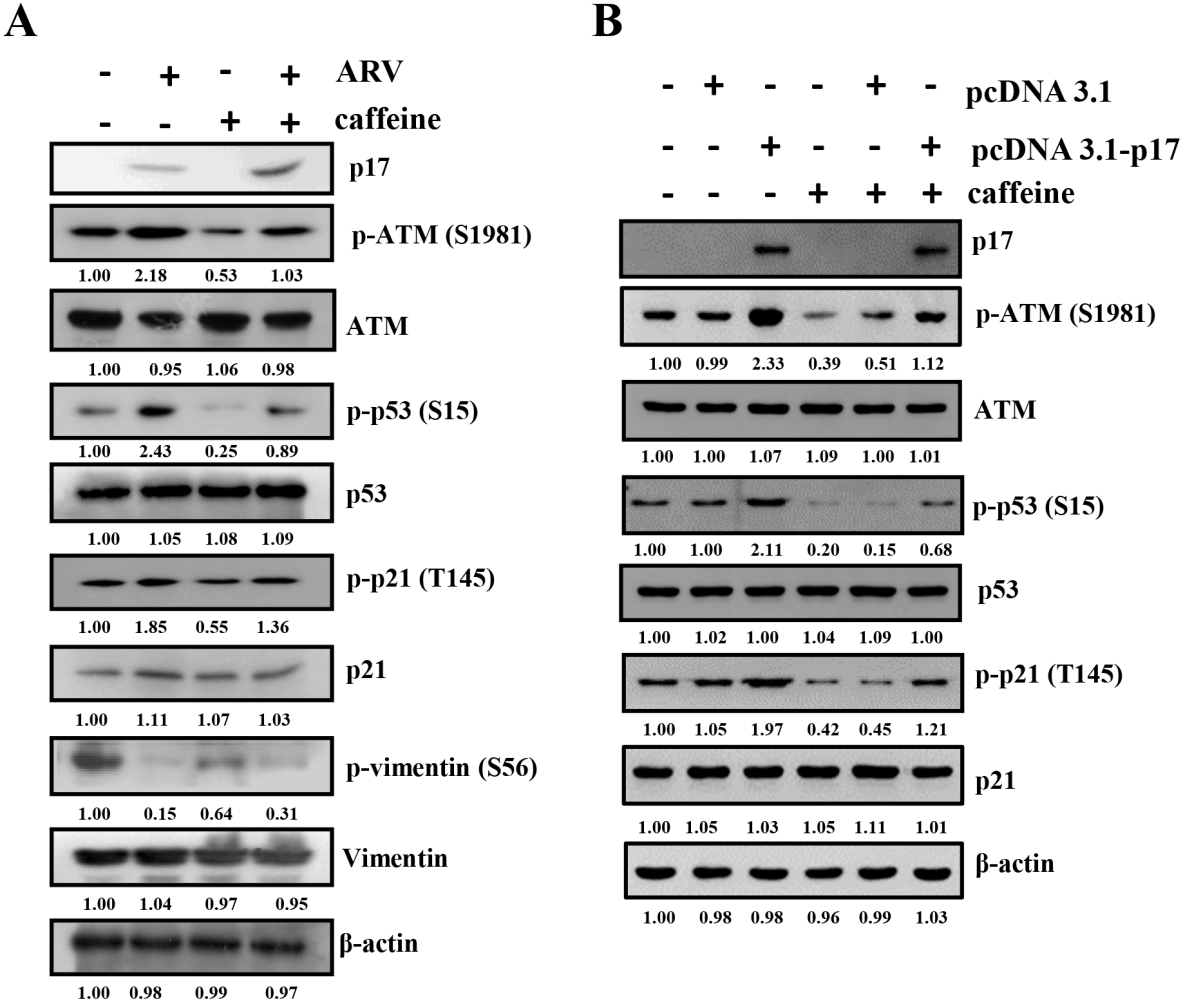
p17 activates p53 and p21 through activation of ATM. To examine the effect of ATM on p53, p2 which include direct interaction with CDK1, p17-mediated suppression of which include direct interaction with CDK1, p17-mediated suppression of 1, and vimentin, Vero cells were pretreated with caffeine (2 mM) for 1h, and then either infected with ARV at a MOI of 5 (A) or transfected with p17 (B) for 24 h. Cell lysates were collected and phosphorylation and protein levels were analyzed by Western blot assays with the respective antibodies. The protein levels were normalized to that for β-actin. The activation and inactivation folds indicated below each lane were normalized against the values for mock-infection or mock-transfection. The levels of the indicated proteins in mock controls were considered 1-fold. Experiments were repeated three times, and representative blots are shown. The uncropped blots with molecular weights are shown in S12 Fig.

Consistent with our previous study [10], a dramatic decrease in the levels of nucleoporin Tpr was observed in both ARV-infected and p17-transfected cells (Fig 6A and 6B). The elevation of p-p53 and p-p21 as well as decrease in the phosphorylation of vimentin at Ser 56 was also seen in both ARV-infected and p17-transfected Vero cells (Fig 6A and 6B). An earlier report has suggested that the increased phosphorylation of ATM at S1981 could activate a G2 checkpoint kinase [32]. Phosphorylation of p53 at S15, a widely accepted target of ATM kinase [33], was detected at 12 hours post infection and post transfection (Fig 6A and 6B) consistent with the known phenomenon that phosphorylation of p53 at S15 is dependent on ATM (Fig 6A and 6B), which suggests an ATM-related response is involved in our experimental settings. Since p21 is one of the downstream targets of p53 [34], p17-mediated activation of p21 is due to the activation of p53 [10]. Therefore, activation of p21 might inhibit the cell cycle. Furthermore, the phosphorylated forms of ATM (S1981), p53 (S15) and p21 (T145) were diminished upon caffeine treatment in ARV-infected and p17-transfected Vero cells (S4 Fig and Fig 7A and 7B). The findings from our experiments suggest that ATM is an upstream kinase of p53. Conversely, the phosphorylated form of vimentin (Ser 56) was restored partially in caffeine-treated cells (Fig 7A), suggesting that in addition to the ATM/Chk1/CDC25C pathway, the ATM/p53 pathway is involved in regulating CDK1and vimentin phosphorylation.

### p17-Mediated Suppression of Plk1 Requires Inactivation of Tpr and Activation of p53 and PP2A

**Fig 8:**
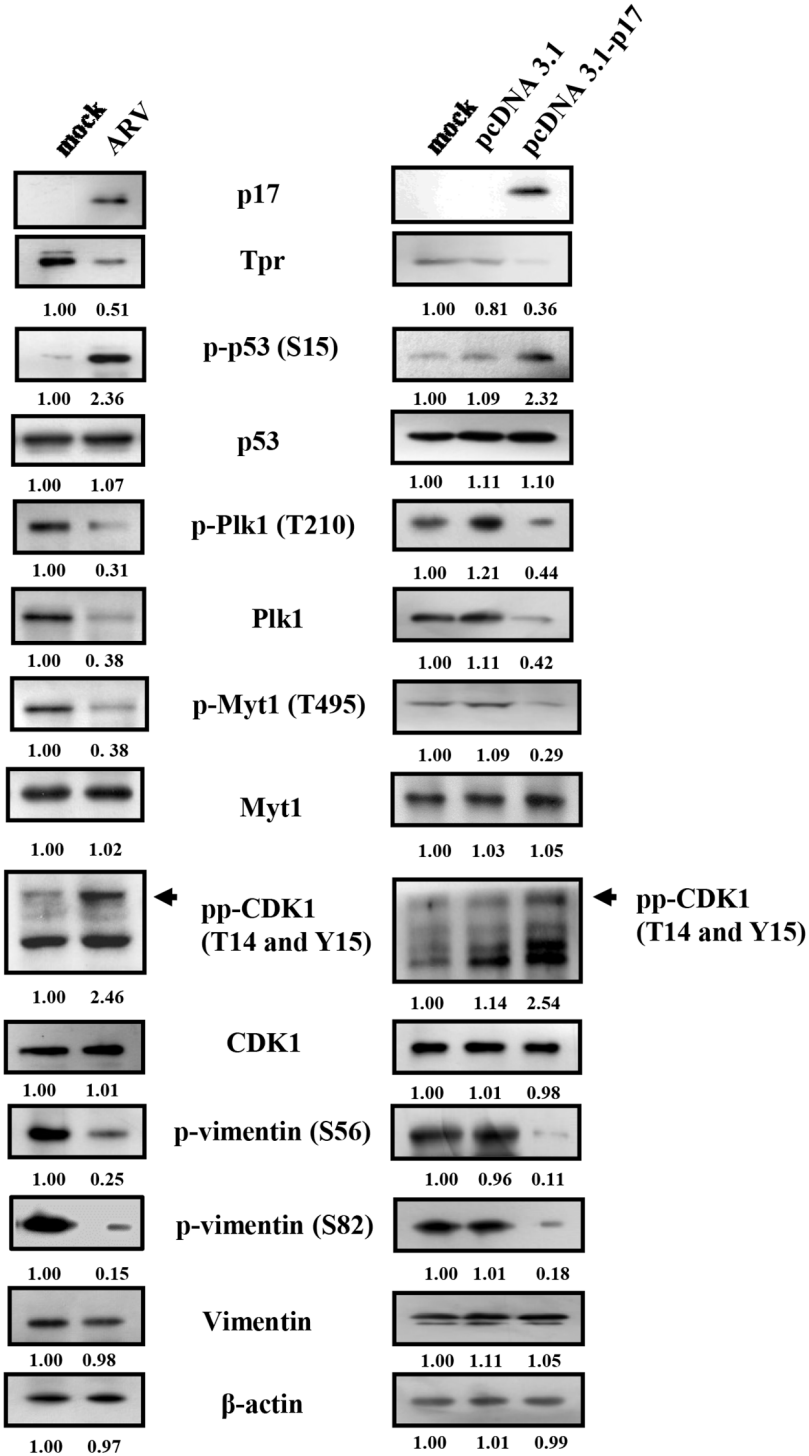
p17 inhibits CDK1 and vimentin phosphorylation at Ser 56 and Ser 82 by p53/Plk1pathways. The levels of Tpr, p-p53, Plk1, p-Plk1 and cell cycle regulatory proteins were examined in ARV-infected (left panel) and p17-transfected (right panel) vero cells. ARV infection and p17 transfection increased the phosphorylated form of CDK1 and reduced the phosphorylated forms of vimentin at Ser 56 and Ser 82. Vero cells were mock-infected, ARV-infected with a MOI of 5, and p17-transfected for 24 h. Cell lysates were collected and phosphorylation and protein levels were analyzed by Western blot assays with the respective antibodies. The protein levels were normalized to that for β-actin. The fold activation and inactivation indicated below each lane were normalized against the values for mock-infection or mock-transfection. The levels of the indicated proteins in the mock controls were considered 1-fold. The experiments were repeated three times, and representative blots are shown. The uncropped blots with molecular weights are shown in S12 Fig.

To further confirm whether the Tpr/p53 pathway plays an important role in regulating Plk1 and CDK1, two independent sets of Vero cells were either infected with ARV at a MOI of 5 or transfected with pcDNA3.1-p17 plasmid for 24 h. Cell lysates were collected and analyzed with respective antibodies. Data presented in this study reveal that a decrease in the Tpr level and a decrease in the phosphorylated form of vimentin (Ser 56) as well as an increase in phosphorylation form of p53 and p21were seen in both ARV-infected and p17-transfected Vero cells (Fig 6A and 6B). A similar trend was also seen in DF-1 cells [10]. To investigate whether p17-mediated inactivation of Plk1 is dependent on the Tpr/p53 and ATM/PP2A signaling pathways, the levels of Tpr, p-p53, p-Plk1, Plk1, p-Myt1, p-CDK1, and p-vimentin were examined. As shown in Fig 8, the phosphorylated forms of p53 and CDK1 were elevated while the phosphorylated forms of Plk1 (T210), Myt1 (T495) and vimentin (Ser 56 and Ser 82) were diminished. Consistent with a recent report suggesting that p53 binds directly to the Plk1 promoter and inhibits Plk1 transcription and translation [11], we found that both phosphorylated Plk1 and Plk1 protein were abolished in ARV-infected and p17-transfected Vero cells (Fig 8). To further confirm the contribution of the Tpr/p53 pathway towards downregulation of Plk1, silencing of both Tpr and p53 was carried out in p17-transfected Vero and DF-1 cells, respectively. As shown in S5 Fig, knockdown of Tpr increased phosphorylated p53 but decreased the level of Plk1, while depletion of p53 restored the levels of Plk1, p-Plk1 and p-vimentin in both DF-1 and Vero cells. Our study identifies a Tpr/p53-dependent pathway that is involved in p17-mediated Plk1 inhibition.

Plk1 has been reported to be phosphorylated *in vivo* at T210 in mitosis and DNA damage prevents phosphorylation at these sites [35]. Since p17 mediates dephosphorylation of Plk1, an in-depth understanding of the mechanisms underlying p17-mediated dephosphorylation of Plk1 will provide additional insights into the biological significance of this effect during virus-host interactions. Moreover, PP2A is targeted by a number of other viral proteins [36]. To assess whether p17 mediates dephosphorylation of Plk1 by activating PP2A, pretreatment with PP2A inhibitor okadaic acid was carried out. As presented in. S6A and S6B Fig, okadaic acid reversed the p17-mediated inhibitory effect of Plk1 phosphorylation (T210) in both ARV-infected and p17-transfected Vero cells, suggesting that dephosphorylation of Plk1 is dependent on PP2A. More recently, the study by Palii and colleagues has demonstrated that activation of PP2A is triggered by the ATM/Chk1 pathway [37]. To further explore whether ATM/Chk1 signaling regulates PP2A, Plk1, and Myt1, we next examined the levels of p-ATM, p-Chk1/2, p-Plk1 and p-Myt1 in okadaic acid- or caffeine-treated cells. As shown in S4 and S7 Figs, decreases in the levels of p-ATM and p-Chk1/2 and increases in the phosphorylated forms of Plk1 and Myt1 were seen in caffeine-treated cells. Caffeine reversed the p17-mediated inhibitory effect of Plk1 phosphorylation (T210), suggesting that ATM mediates dephosphorylation of Plk1. The decrease in phosphorylated Plk1 was seen in okadaic acid-treated cells and its phosphorylation levels could be reversed in ARV-infected and p17-transfected cells (S6A and S6B Fig). Based on our findings, we conclude that dephosphorylation of Plk1 by PP2A is dependent on the ATM/Chk1signal pathway.

It was demonstrated that the activity of CDK1 is regulated by the phosphorylation status of tyrosine 14 and 15 on CDK1 [2], which is phosphorylated by Myt1 during late G2 and is rapidly dephosphorylated by the CDC25C phosphatase to trigger entry into mitosis. Because Plk1 phosphorylation of Myt1 at T495 has been proposed to inactivate Myt1, one of the kinases known to phosphorylate CDK1 at T14/Y15 [2], we thus examined the level of phosphorylated Myt1 (T495) in okadaic acid-treated cells. Findings from the present study indicate that decreased levels of phosphorylated Myt1 (T495) in both ARV-infected and p17-transfected cells were reversed in okadaic acid-treated groups (S6A and S6B Fig). The results further confirm that the p17 protein is able to activate Myt1 by suppressing Plk1 through activation of both PP2A and p53, thereby blocking CDK1 kinase activity by the phosphorylation of two conserved residues (T14 and Y15).

### Blockade of the ATM-Dependent Pathway Reverses Phosphorylation of Vimentin at Ser 56 and Ser 82

To further confirm that ATM is the upstream signal for p17-mediated phosphorylation of vimentin at Ser 56 and Ser 82, Vero cells were pretreated with caffeine (2 mM) for 2 hours, followed by infection with ARV S1133 at a MOI of 5 for 24 hr. Cell lysates were then analyzed with the indicated antibodies. Results reveal decrease in the p-ATM, p-p53 and p-CDK1 levels and an increase in the p-Myt1 in caffeine-treated Vero cells as compared to ARV-infected cells (S7 Fig). The significant decreases in p-Plk1, Plk1, and p-vimentin levels were observed in ARV-infected cells. The effect of ARV p17 on phosphorylated levels of Plk1, Myt1, CDK1, and vimentin (Ser 56 and Ser 82) could be reversed in caffeine-treated cells (S7 Fig). Taken together, our results further confirm that p17-mediated suppression of vimentin phosphorylation is ATM-dependent. Original images of all gels and blots with molecular weight are shown in S9, S10, S11, S12 and S13 Figs.

### ARV Infection and p17 Transfection Result in Cell Growth Retardation and Accumulation of Cells in the G2/M Phases

**Fig 9:**
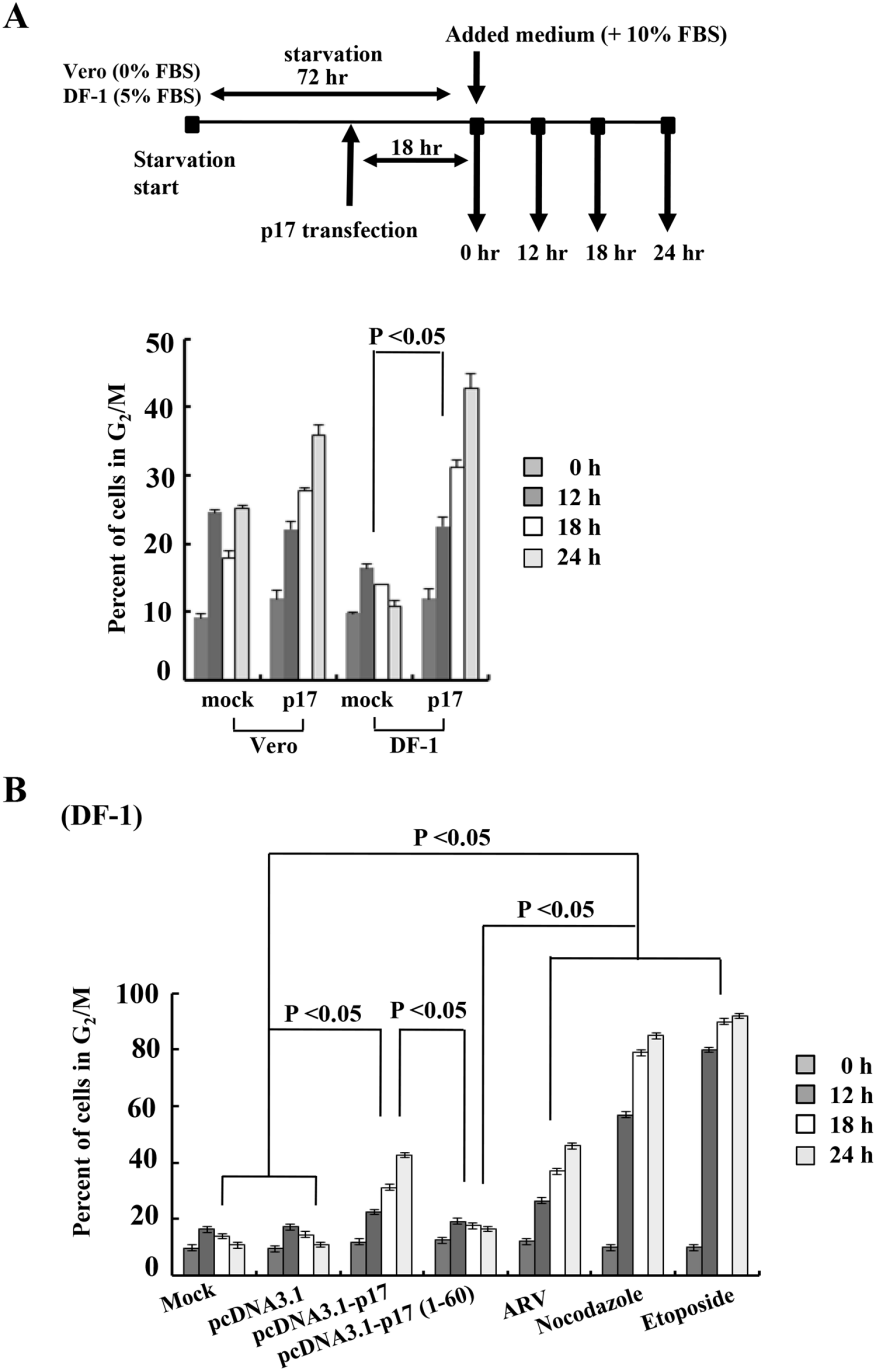
ARV infection and p17 transfection result in cell growth retardation and accumulation of cells in the G2/M phases. (A) As described in Material and Methods, both DF-1 and Vero cells were G0/G1 phase synchronized using serum deprivation by maintenance of the cells in either DMEM containing no FBS (for Vero cells) or 5% FBS (for DF-1 cells) supplementation for 72 h. Cells were transfected for 18 h with constructs (p17 and vector only) after serum deprivation for 54 h. The experimental design for G0/G1 phase synchronization using serum deprivation is shown (upper panel). The percentages of Vero or DF-1cells accumulating in the G2/M phase of the cell cycle at different time points are shown. (B) The percentages of DF-1cells accumulating in the G2/M phase of the cell cycle at different time points. DF-1 cells infected with ARV at a MOI of 5 as well as p17-, p17(1–60)-, and pcDNA3.1-transfected, and chemical-treated cells at different time points are shown. The chemicals were dissolved in DMSO. Results representative of three independent experiments are shown. The results are presented as the mean of three independent experiments.

Having demonstrated that the ARV p17 protein inhibits CDK1 kinase activity leading to suppression of vimentin phosphorylation at Ser 56 and Ser 82, we next wanted to identify the phase in the cell cycle at which p17 inhibits cellular proliferation in both ARV-infected and p17-transfected DF-1 and Vero cells using flow cytometry. We found that ARV-infection and p17-transfection resulted in cell growth retardation and an increase in the percentage of DF-1 and Vero cells in G2/M phases (Fig 9A and 9B) compared to mock-infected cells at different time points. The G2/M phase arrest was also observed in p17-transfected DF-1 and Vero cells in a time-dependent manner (Fig 9A). Importantly, the p17-(1–60) mutant that loses its CDK1 binding activity (Fig 1C) did not cause retardation of the cell cycle or G2/M cell cycle arrest (Fig 9B). Representative cell cycle profiles of DF-1 and Vero cells transfected by p17 are shown in S8 Fig. Interestingly, in addition to inducing G2/M cell cycle arrest, p17 may retard the cell cycle at G0/G1 or S phases (S8 Fig). This finding is supported by our recent study suggesting that p17 causes a down-regulation of CDK4 and cyclin D1 and upregulation of p21, a CDK inhibitor [10]. The precise mechanisms need to be further explored. In this work, more than 80% of cells treated with nocodazole or etoposide were arrested in G2/M phase 24 hours post treatments (Fig 9B). Our results reveal that ARV p17 retards the cell cycle and results in accumulation of cells in the G2/M phases.

### Suppression of Tpr and CDK1 Increases Virus Replication While Blockade of ATM, p53, and Vimentin Reducing Virus Replication

**Fig 10:**
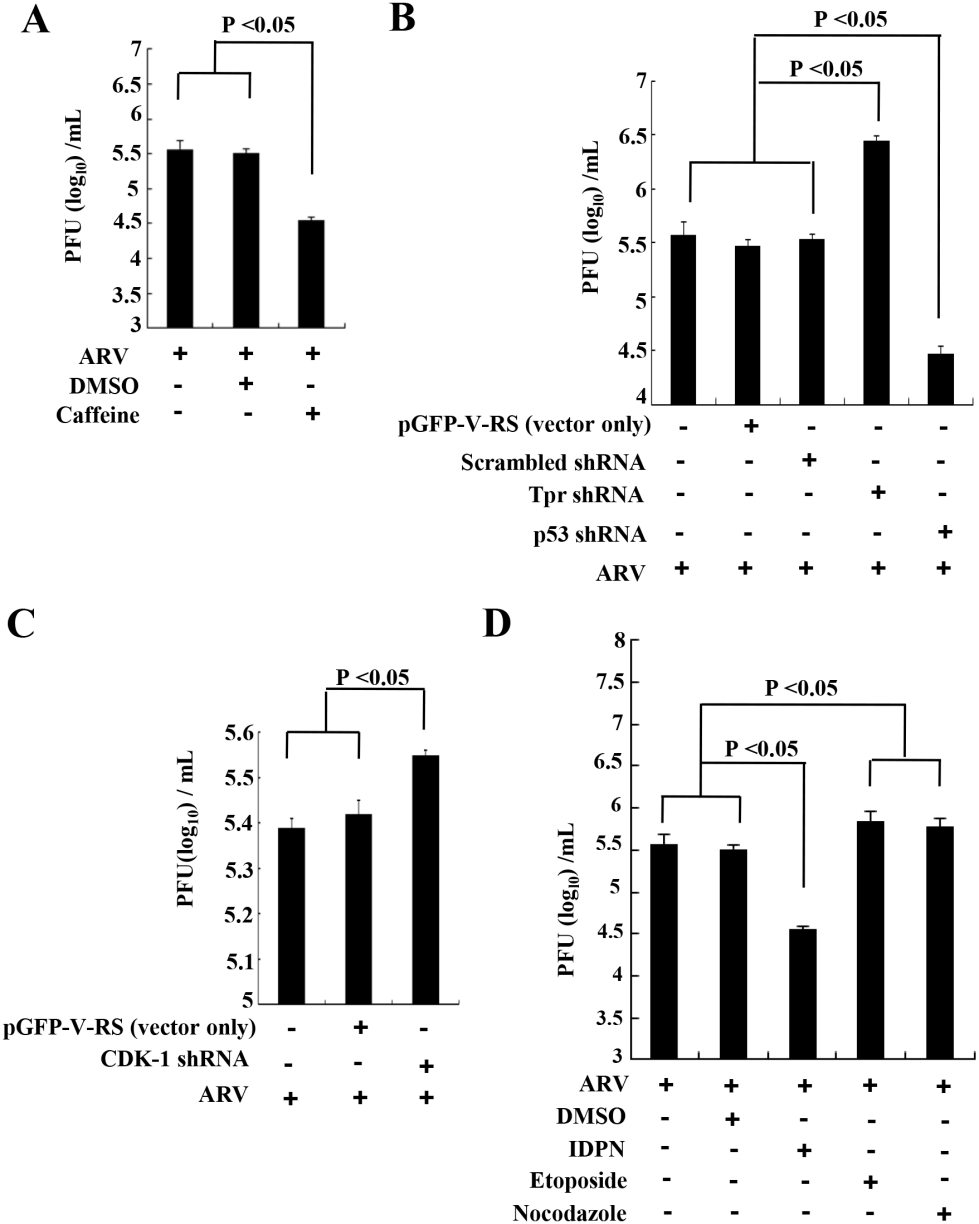
Suppression of Tpr, CDK1 and vimentin is beneficial for virus replication. (A) To determine the role of ATM in ARV replication, treatment with caffeine (2 mM) was carried out in ARV-infected Vero cells. The supernatants of treated-cells in each well were harvested at 24 hpi for viral titration. (B-D) To determine the effect of Tpr, p53 (B), CDK1(C), and vimentin (D) on ARV replication, depletion of Tpr, p53, and CDK1 with respective shRNAs and inhibition of vimentin by IDPN (1%) were carried out in ARV-infected Vero cells. The effects of G2/M cell cycle inhibitors nocodazole (60 ng/ mL) and etoposide (2 uM) on ARV replication were also examined (D). The supernatants of treated-cells in each well were harvested at 24 hpi for viral titration. All data shown represent the mean±SD calculated from three independent experiments.

To investigate the role of ATM, Tpr, p53, CDK1, and vimentin in ARV replication, knockdown of Tpr, p53, and CDK1 with shRNAs as well as inhibition of ATM and vimentin by caffeine and IDPN, respectively were carried out in ARV-infected Vero cells. In this work, knockdown of p53 as well as inhibition of ATM and vimentin by inhibitors diminished virus yield (Fig 10A, 10B and 10D). Conversely, knockdown of Tpr and CDK1 by shRNAs increased virus yield (Fig 10B and 10C). Further, in the positive control groups, we also observed that ARV titers in Vero cells treated with G2/M-phase-arrest-inducing drugs nocodazole or etoposide, respectively also increased in G2/M synchronized Vero cells (Fig 10D). The effects of Tpr and p53 on virus replication are consistent with our earlier studies [10, 19]. Our results show how ARV p17 modulates ATM, nucleoporin Tpr, and p53 that in turn block cdc25C, Plk1, and CDK1 leading to suppression of vimentin phosphorylation which causes G2/M cell cycle arrest and benefits virus replication.

## Discussion

ARV, like other viruses, has evolved strategies that alter the physiology of the host cells during viral replication to promote its replication. This work was an extension of our earlier studies in which it was discovered that ARV p17 induces cell growth retardation and influences phosphorylation of elongation and initiation factors and host protein translation [10, 17, 18]. However, the precise mechanism(s) by which ARV negatively controls cell growth remains unclear. In this work, the novel discovery that p17 functions as a suppressor of both CDK1 and Plk1 offers important insight into its functionality in modulating the G2/M cell cycle. In this report, detailed studies were undertaken to define regions involved in the interaction of p17 with CDK1 or vimentin. We demonstrate for the first time that p17 directly interacts with CDK1 or vimentin which in turn suppresses the CDK1-vimentin enzyme-substrate reaction, thereby blocking the binding of the PBD of Plk1 to phosphorylated vimentin-Ser56 and impacting subsequent vimentin phosphorylation at Ser 82 by Plk1. This study also provides strong evidence that the ARV p17 protein elicits a cellular response that involves activation of multiple upstream signaling pathways, thereby negatively regulating PlK1, CDK1, and vimentin, causing G2/M cell cycle arrest. However, experiments for exploring other crucial targets for delaying other phases of the cell cycle are underway.

Several viruses are influenced by or alter CDK1 kinase activity [38–42]. In cases where viruses increase CDK1 kinase activity, it is possible that this activity is essential for phosphorylating viral proteins. For example, herpes simplex virus ICP0 [41], Epstein-Barr virus EBNA-LP [43], hepatitis E virus ORF3 [44] and varicella-zoster virus glycoprotein gI are phosphorylated by CDK1 [45]. In contrast, human papillomavirus E2, human immunodeficiency virus (HIV) Vpr, and reovirus σ1s inhibit or delay the activation of CDK1 [36, 42,46, 47]. These observations suggest that viruses have evolved mechanisms to alter CDK1 function. Furthermore, in many viruses, such as HIV Vpr and reovirus σ1s, CDK1 inhibition by suppressing CDC25C and subsequent G_2_/M cell cycle arrest result in increased viral replication [42, 48, 49]. In this work, the mechanism underlying p17-mediated inhibition of CDK1 kinase activity and subsequent suppression of vimentin phosphorylation has been elucidated. A compelling finding from the present study is that p17 negatively regulates Plk1 by suppressing Tpr and by activating the ATM-, p53-, and PP2A-dependent pathways. This is consistent with a previous study suggesting that inactivation of Plk1 by ATM and PP2A following DNA damage, thereby leading to arrest at the G2/M boundary [49].

Earlier studies suggested that both HIV Vpr and reovirus σ1s could inhibit CDK1 activity via inactivation of CDC25C [42, 47]. Recently, Davy and colleagues proposed a different mechanism that inhibits CDK1 function. This group found that human papillomavirus type 16 E1-E4-induced G2 arrest is associated with cytoplasmic retention of active CDK1/cyclin B1 complexes [50]. Conversely, an increase in CDK1 activity during the G2/M phase was seen in herpes simplex virus 1-infected cells [41]. Importantly, ARV p17 seems to use different strategies to inhibit CDK1 function. This study provides two lines of evidence for p17-mediated inhibition of CDK1 function. Firstly, p17 binds to and inhibits the CDK1 kinase activity. Secondly, p17 causes inhibitory phosphorylation of CDK1 by suppressing both Plk1 and CDC25C. Earlier studies have demonstrated that CDC25C activity is inhibited following phosphorylation by the kinases Chk1/2 [26–28]. In the current and our previous studies [25], we have demonstrated that ARV p17 upregulates Chk1/2 in an ATM-dependent fashion. It is possible that p17 is the major viral protein that is involved in inducing a checkpoint pathway in response to DNA replication stress or a DNA damage-like response [25].

Several previous studies have suggested that vimentin plays an important role in several virus infections [51–58]. In this work, we found that inhibition of vimentin by IDPN diminished virus yield, implying that vimentin plays a role in virus replication. Although we have demonstrated that ARV entry follows a caveolin-1-mediated and dynamin-2-dependent endocytic pathway [14], the receptor(s) that are necessary for ARV entry remain unknown. Experiments for exploring whether vimentin serves as a receptor for ARV entry are underway. During infection with retroviruses such as bovine leukemia virus (BLV) and HIV, the viral-encoded protease specifically cleaves vimentin. Surface-expressed vimentin is necessary for cell entry of several viruses [56, 57]. Investigations of other viruses have shown that viral proteins directly bind vimentin as observed with dengue virus nonstructural protein 1, where vimentin binding is critical for virus replication [53], and in bluetongue virus, the binding of VP2 to vimentin is necessary for viral egress [51]. In the case of African swine fever virus (ASFV), it has been shown that vimentin is arranged around viral factories, forming a cage-like structure, which may aid in isolation of viral proteins from the rest of the cell [54, 56]. The study by Gladue et al. suggested that a cage-like structure around Foot-and-mouth disease virus (FMDV) protein 2C was seen, and this cage-like structure disappeared as viral infection progressed [58]. The precise mechanism of vimentin that influences ARV replication remains to be explored.

Growing evidence indicates that viral infection, viral protein expression, or the presence of viral DNA causes the host cell to arrest at G2/M phase to generate a favorable environment for viral replication [48, 49, 59, 60]. Thus, an in-depth understanding of the molecular mechanisms underlying ARV p17 protein-induced G2/M-phase cell cycle arrest will provide additional insights into the basic biology of G2/M-phase cell cycle regulation and the biological significance of this effect during ARV-host interactions. In this study, we show that ARV, a cytoplasmic virus, has two viral proteins (p17 and σA) targeting to the nucleus [16, 61]. By shuttling to the nucleus, p17 functions as a Tpr suppressor, leading to activating p53, p21, and PTEN [10]. Thus, p17 exerts its effect on nuclear signaling pathways. Data from the present study reveal that ARV p17 induces G2/M-phase cell cycle arrest by blocking both CDK1 and Plk1 function through interacting with CDK1 or by through activation of the Tpr/p53/p21 pathway, leading to suppression of phosphorylation of vimentin at Ser 56 and Ser 82. Knockdown of p53 with shRNA or inhibition of ATM by caffeine all reduced virus yield while depletion of CDK1 and Tpr with shRNAs increased virus production. Our recent study revealed that depletion of CDK4 with shRNAs also increased virus production [10]. These results imply that p17-mediated G2/M-phase cell cycle arrest or G0/G1 cell cycle arrest in replication-activated cells may allow the virus to access the host replication machinery without competing with cellular DNA replication.

**Fig 11:**
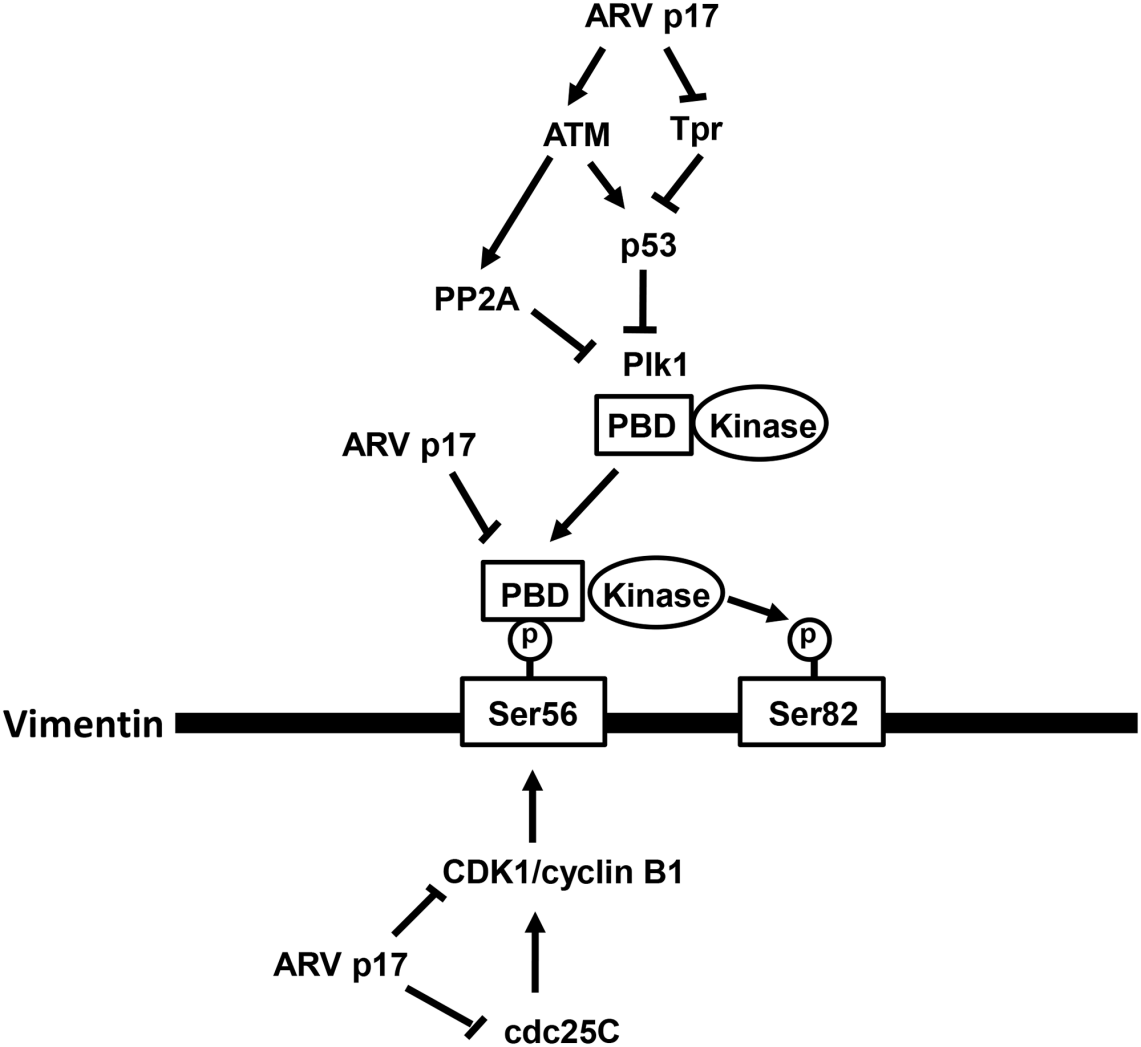
A proposed model depicting the molecular mechanism of ARV p17 regulating ATM, Tpr, p53, CDC25C, Plk1, and CDK1 to mediate vimentin phosphorylation to control cell cycle progression. p17 suppresses Tpr and activates ATM leading to p53 activation, which in turn activates p21 and inactivates Plk1 and CDC25C. The outcome of this is to reduce vimentin phosphorylation at Ser 56 and Ser 82. p17 inhibits CDC25C through activation of ATM/chk1. CDC25C, a dual-specificity phosphatase, dephosphorylates CDK1 (T14 and Y15) at two phosphorylation sites for CDK1 activation. In addition, p17 blocks Plk1 by activating p53 to inhibit Plk1 gene expression and by activating PP2A to dephosphorylate Plk1, thereby reducing vimentin phosphorylation at Ser 56 and Ser 82. p17 interacts directly with CDK1 and vimentin-(45–65) which inhibits the CDK1-vimentin enzyme-substrate reaction, thereby blocking the binding of PBD of Plk1 to phosphorylated vimentin-Ser 56 and subsequent vimentin phosphorylation at Ser 82 by Plk1, which cause G2/M cell cycle arrest.

A clearer understanding of the molecular basis for virus-induced changes can shed light on normal cellular events as well as on the specific mechanisms that ARV uses to gain control over its host. A schematic model shown in Fig 11 depicts the mechanisms underlying ARV p17 regulating ATM, Chk1/2, Tpr, p53, Plk1, CDC25C, CDK1, and vimentin to control cell cycle progression. This study also elucidates the mechanisms of p17-mediated blockade of CDK1-vimentin enzyme-substrate reactions and suppression of binding of the PBD of Plk1 to phosphorylated vimentin-Ser 56 and subsequent vimentin phosphorylation at Ser 82 by Plk1. The present work demonstrates for the first time that direct interaction of p17 with CDK1 and a regulatory network of p17 linking ATM, chk1/2, CDC25C, Tpr, p53, p21, and Myt-1 lead to suppression of Plk1 and CDK1 and blockade of vimentin phosphorylation at Ser 56 and Ser 82, both of which cause G2/M cell cycle arrest and benefit virus replication.

## Supporting Information

S1 Fig Expression and purification of GST, GST-p17, GST-CDK1, GST-vimentin, GST-cyclin B1, and TrxA-His-p17. The p17 gene of ARV S1133 strain, CDK1, cyclin B1, and vimentin genes were amplified by PCR using primer pairs as indicated in Table 2. As mentioned in Material and Method section, the amplified PCR products were cut with respective restriction enzymes and then introduced into the corresponding sites in either pET32a or pGEX4T-1 vectors. The procedures for expression and purification of these proteins are described in the Material and Method section. The purified proteins were electrophoresed in 12% polyacrylamide gels at 70 V through the stacking gel and at 100 V through the resolving gels. The uncropped gels with molecular weights are shown in S9 Fig.
(TIF)

S2 Fig p17 co-localizes with cellular vimentin. Vero cells were transfected with pcDNA3.1-p17 plasmid for 24 hours, followed by immunofluorescence staining by the indicated antibodies. Colocalization of p17 and vimentin was visualized by immunofluorescence staining.
(TIF)

S3 Fig p17 mediates suppression of CDK1/cyclin B1 complex kinase activity. To examine whether p17 interacts with the CDK1/cyclin B1 complex leading to inhibition of CDK1 kinase activity and vimentin phosphorylation at Ser 56, an *in vitro* kinase assay using GST-vimentin as a substrate was performed. TrxA-His-p17 and GST-vimentin were added after 30 min incubation of GST-CDK1 and GST-cyclin B1proteins.
(TIF)

S4 Fig The inhibitory effect of caffeine on ATM, and Chk1/Chk2. Vero cells were pretreatment with caffeine (2 mM) for 1h, followed by infection with ARV at a MOI of 10 (A) or transfection with pcDNA3.1-p17 plasmid (B) for 24 h. Cell lysates were collected and analyzed by Western blot assays with the indicated antibodies. Experiments were repeated three times, and representative blots are shown.
(TIF)

S5 Fig Knockdown of Tpr activated p53 leading to suppression of Plk1 and vimentin. Vero (left panel) and DF-1 cells (right panel) were co-transfected with pcDNA3.1-p17, Tpr shRNA, p53 shRNA, scramble shRNA, and pGFP-V-RS (vector only), respectively, for 24 hours. The expression levels of indicated proteins were examined in p17and Tpr shRNA-co-transfected cells as well as p17 and p53 shRNA-cotransfected cells. The phosphorylated forms of p53, Plk1 and vimentin were analyzed by Western blot assays with the indicated antibodies. Cell lysates were collected and phosphorylation and protein levels were analyzed by Western blot assays. The protein levels were normalized to that for β-actin. The fold activation and inactivation indicated below each lane were normalized against the values for mock-transfection. The levels of the indicated proteins in the mock controls were considered 1-fold. The uncropped blots with molecular weights are shown in S10 Fig.
(TIF)

S6 Fig PP2A inhibitor okadaic acid reverses the p17-mediated inhibitory effect of PlK1 phosphorylation. Vero cells were pretreatment with PP2A inhibitor okadaic acid (100 nM) for 1h, followed by infection with ARV at a MOI of 10 (A) or transfection with pcDNA3.1-p17 plasmid (B) for 24 h. The phosphorylated forms of p-Plk1 (T210) and p-Myt1 (T495) were analyzed by Western blot assays with the indicated antibodies. The protein levels were normalized to that for β-actin. The fold activation and inactivation indicated below each lane were normalized against the values for mock-infection or mock-transfection. The levels of the indicated proteins in the mock controls were considered 1-fold. Experiments were repeated three times, and representative blots are shown. The uncropped blots with molecular weights are shown in S10 Fig.
(TIF)

S7 Fig Blockade of ATM with caffeine restores phosphorylation of Plk1 and vimentin at Ser 56 and Ser 82 in ARV-infected Vero cells. Vero cells were pretreated with caffeine (2 mM) for 1h, followed by infection with ARV at a MOI of 10 (A) or transfection with pcDNA3.1-p17 plasmid (B) for 24 h. Cell lysates were collected and analyzed by Western blot assays with the indicated antibodies. The protein levels were normalized to that for β-actin. The fold activation and inactivation indicated below each lane were normalized against the values for mock-infection. The levels of the indicated proteins in the mock controls were considered 1-fold. Experiments were repeated three times, and representative blots are shown. The uncropped blots with molecular weights are shown in S10 Fig.
(TIF)

S8 Fig Representative cell cycle profiles of DF-1 and Vero cells transfected by p17. The phases in the cell cycle at which p17 inhibits cellular proliferation in both p17-transfected DF-1 and Vero cells by using flow cytometry are shown. Vero cells require 16 hours to complete a round of cell cycle while DF-1 cells need 24 hours.
(TIF)

S9 Fig Original images of blots with molecular weights (KDa). (TIF)

S10 Fig Original images of blots with molecular weights (KDa). (TIF)

S11 Fig Original images of blots with molecular weights (KDa). (TIF)

S12 Fig Original images of gels and blots with molecular weights (KDa). (TIF)

S13 Fig Original images of blots with molecular weights (KDa). (TIF)

## References

[1] Jackman MR, Pines JN (1997)See comment in PubMed Commons belowCyclins and the G2/M transition. Cancer Surv 29: 47–73. 9338096

[2] Fattaey A, Booher RN (1997) Myt1: a Wee1-type kinase that phosphorylates Cdc2 on residue Thr14. Prog. Cell Cycle Res 3: 233–240. 9552418 10.1007/978-1-4615-5371-7_18

[3] Kotani S, Tugendreich S, Fujii M, Jorgensen PM, Watanabe N, Hoog C, et al (1998) PKA and MPF-activated polo-like kinase regulate anaphase- promoting complex activity and mitosis progression. Mol Cell 1: 371–380. 9660921 10.1016/s1097-2765(00)80037-4

[4] Hamanaka R, Smith MR, O'Connor PM, Maloid S, Mihalic K, Spivak JL, et al (1995) Polo-like kinase is a cell cycle-regulated kinase activated during mitosis. J Biol Chem 270: 21086–21091. 7673138 10.1074/jbc.270.36.21086

[5] Yamaguchi T, Goto H, Yokoyama T, Silljé H, Hanisch A, Uldschmid A, et al (2005) Phosphorylation by Cdk1 induces Plk1-mediated vimentin phosphorylation during mitosis. J Cell Biol 171(3): 431–436. 16260496 10.1083/jcb.200504091 PMC2171270

[6] Yu J, Fleming SL, Williams B, Williams EV, Li Z, Somma P, et al (2004) Greatwall kinase: a nuclear protein required for proper chromosome condensation and mitotic progression in Drosophila. J Cell Biol 164: 487–492. 14970188 10.1083/jcb.200310059 PMC2171981

[7] van Vugt MA, Smits VA, Klompmaker R, Medema RH (2001) Inhibition of Polo-like kinase-1 by DNA damage occurs in an ATM- or ATR-dependent fashion. J Biol Chem 276: 41656–41660. 11514540 10.1074/jbc.M101831200

[8] Frosst P, Guan T, Subauste C, Hahn K, Gerace L (2002) Tpr is localized within the nuclear basket of the pore complex and has a role in nuclear protein export. J Cell Biol 156: 617–630. 11839768 10.1083/jcb.200106046 PMC2174070

[9] Funasaka T, Tsuka E, Wong RW (2012) Regulation of autophagy by nucleoporin Tpr Sci Rep 2: 878–886. 10.1038/srep00878 23170199 PMC3501823

[10] Huang WR, Chiu HC, Liao TL, Chuang KP, Shih WL, Liu HJ (2015) Avian reovirus protein p17 functions as a nucleoporin Tpr suppressor leading to activation of p53, p21 and PTEN and inactivation of PI3K/AKT/mTOR and ERK signaling pathways. PLOS One 10(8): e0133699 10.1371/journal.pone.0133699 26244501 PMC4526660

[11] McKenzie L, King S, Marcar L, Nicol S, Dias SS, Schumm K, et al (2010) p53-dependent repression of polo-like kinase-1 (PLK1). Cell Cycle 9(20): 4200–4212. 20962589 10.4161/cc.9.20.13532 PMC3055203

[12] Fuchs E, Weber K (1994)Intermediate filaments: structure, dynamics, function, and disease. Annu Rev Biochem 63: 345–382. 7979242 10.1146/annurev.bi.63.070194.002021

[13] Spandidos DA, Graham AF (1976) Physical and chemical characterization of an avian reovirus. J Virol 19: 968–976. 987252 10.1128/jvi.19.3.968-976.1976 PMC354937

[14] Huang WR, Wang YC, Chi PI, Wang L, Wang CY, Lin CH, et al (2011) Cell entry of avian reovirus follows a caveolin-1-mediated and dynamin-2- dependent endocytic p athway that requires activation of p38 MAPK and Src signaling pathways as well as microtubules and small GTPase Rab5 protein. J Biol Chem 286: 30780–30794. 10.1074/jbc.M111.257154 21705803 PMC3162439

[15] Shmulevitz M, Yameen Z, Dawe S, Shou J, O'Hara D, Holmes I, et al (2002) Sequential partially overlapping gene arrangement in the tricistronic S1 genome segments of avian reovirus and Nelson Bay reovirus: implications for translation initiation. J Virol 76: 609–618. 11752152 10.1128/JVI.76.2.609-618.2002 PMC136829

[16] Costas C, Martinez-Costas J, Bodelon G, Benavente J (2005) The second open reading frame of the avian reovirus S1 gene encodes a transcription- dependent and CRM1-independent nucleocytoplasmic shuttling protein. J Virol 79: 2141–2150. 15681417 10.1128/JVI.79.4.2141-2150.2005 PMC546569

[17] Liu HJ, Lin PY, Lee JW, Hsu HY, Shih WL (2005) Retardation of cell growth by avian reovirus p17 through the activation of p53 pathway. Biochem Biophy Res Commun 336: 709–715. 10.1016/j.bbrc.2005.08.149 PMC7092890 16143310

[18] Ji WT, Wang L, Lin RC, Huang WR, Liu HJ (2009) Avian reovirus influences phosphorylation of several factors involved in host protein translation including eEF2 in Vero cells. Biochem Biophy Res Commun 384: 301–305. 10.1016/j.bbrc.2009.04.116 19406104

[19] Chi PI, Huang WR, Lai IH, Cheng CY, Liu HJ (2013) The p17 nonstructural protein of avian reovirus triggers autophagy enhancing virus replication via activation of PTEN and AMPK as well as PKR/eIF2α signaling pathways. J Biol Chem 288: 3571–3584. 10.1074/jbc.M112.390245 23233667 PMC3561576

[20] Kitagawa M, Higashi H, Jung HK, Suzuki-Takahashi I, Ikeda M, Tamai K, et al (1996) The consensus motif for phosphorylation by cyclin D1-Cdk4 is different from that for phosphorylation by cyclin A/E-Cdk2. EMBO J 15(24): 7060–7069. 9003781 PMC452531

[21] Paull TT, Rogakou EP, Yamazaki V, Kirchgessner CU, Gellert M, Bonner M (2000) A critical role for histone H2AX in recruitment of repair factors to nuclear foci after DNA damage. Curr Biol 10: 886–895. 10959836 10.1016/s0960-9822(00)00610-2

[22] Kastan MB, Lim DS (2000) The many substrates and functions of ATM. Nat. Rev. Mol Cell Biol 1: 179–186. 11252893 10.1038/35043058

[23] Melchionna R, Chen XB, Blasina A, McGowan CH (2000) Threonine 68 is required for radiation-induced phosphorylation and activation of Cds1. Nat Cell Biol 2: 762–765. 11025670 10.1038/35036406

[24] Falck J, Mailand N, Syljuasen RG, Bartek J, Lukas J (2001) The ATM-Chk2- Cdc25A checkpoint pathway guards against radioresistant DNA synthesis. Nature 410: 842–847. 11298456 10.1038/35071124

[25] Lin PY, Liu HJ, Chang CD, Chang CI, Hsu JL, Liao MH, et al (2011) Avian reovirus S1133-induced DNA damage signaling and subsequent apoptosis in cultured cells and in chickens. Arch Virol 156: 1917–1929. 10.1007/s00705-011-1063-3 21779911

[26] Furnari B, Rhind N, Russell P (1997) Cdc25 mitotic inducer targeted by Chk1 DNA damage checkpoint kinase. Science 277: 1495–1497. 9278510 10.1126/science.277.5331.1495

[27] Peng CY, Graves PR, Thoma RS, Wu Z, Shaw AS (1997) Mitotic and G_2_ checkpoint control: regulation of 14-3-3 protein binding by phosphorylation of Cdc25C on serine-216. Science 277: 1501–1505. 9278512 10.1126/science.277.5331.1501

[28] Dalal SN, Schweitzer CM, Gan J, DeCaprio JA (1999) Cytoplasmic localization of human cdc25C during interphase requires an intact 14-3-3 binding site. Mol Cell Biol 19: 4465–4479. 10330186 10.1128/mcb.19.6.4465 PMC104405

[29] Chen F, Zhang Z, Bower J, Lu Y, Leonard SS, Ding M, et al (2002) Arsenite- induced Cdc25C degradation is through the KEN-box and ubiquitin-proteasome pathway. Proc Natl Acad Sci USA 99: 1990–1995. 11842186 10.1073/pnas.032428899 PMC122307

[30] Chen YI, Lin CH, Ji TW, Li SK, Liu HJ (2008) Proteasome inhibition reduces avian reovirus replication and apoptosis induction in cultured cells. J Virol Methods 151: 95–100. 10.1016/j.jviromet.2008.03.016 18455810 PMC7119659

[31] Sanchez Y, Wong C, Thoma RS, Richman R, Wu Z, Piwnica-Worms H, et al (1997) Conservation of the Chk1 checkpoint pathway in mammals: linkage of DNA damage to Cdk regulation through Cdc25. Science 277: 1497–1501. 9278511 10.1126/science.277.5331.1497

[32] Stiff T, Walker SA, Cerosaletti K, Goodarzi AA, Petermann E, Concannon P, et al (2006) ATR-dependent phosphorylation and activation of ATM in response to UV treatment or replication fork stalling. EMBO J 25(24): 5775–5782. 17124492 10.1038/sj.emboj.7601446 PMC1698893

[33] Banin S, Moyal L, Shieh S, Taya Y, Anderson CW, Chessa L, et al (1998) Enhanced phosphorylation of p53 by ATM in response to DNA damage. Science 281: 1674–1677. 9733514 10.1126/science.281.5383.1674

[34] Jung YS, Qian Y, Chen X (2010) Examination of the expanding pathways for the regulation of p21 expression and activity. Cell Signal 22: 1003–1012. 10.1016/j.cellsig.2010.01.013 20100570 PMC2860671

[35] Loncarek J, Hergert P, Khodjakov A (2010) Centriole reduplication during prolonged interphase requires procentriole maturation governed by Plk1. Curr. Biol. 20(14): 1277–1282. 10.1016/j.cub.2010.05.050 20656208 PMC2911792

[36] Hrimech M, Yao XJ, Branton PE, Cohen EA (2000) Human immunodeficiency virus type 1 vpr-mediated G_2_ cell cycle arrest: Vpr interferes with cell cycle signaling cascades by interacting with the B subunit of serine/threonine protein phosphatase 2A. EMBO J 19: 3956–3967. 10921877 10.1093/emboj/19.15.3956 PMC306605

[37] Palii SS, Cui Y, Innes CL, Paules RS (2013) Dissecting cellular responses to irradiation via targeted disruptions of the ATM-CHK1-PP2A circuit. Cell Cycle 12(7): 1105–1118. 10.4161/cc.24127 23462183 PMC3646866

[38] Belyavskyi M, Braunagel SC, Summers MD (1998) The structural protein ODV- EC27 of Autographa californica nucleopolyhedrovirus is a multifunctional viral cyclin. Proc Natl Acad Sci USA 95: 11205–11210. 9736714 10.1073/pnas.95.19.11205 PMC21620

[39] Braunagel SC, Parr R, Belyavskyi M, Summers MD (1998) Autographa californica nucleopolyhedrovirus infection results in Sf9 cell cycle arrest at G_2_/M phase. Virology 244: 195–211. 9581791 10.1006/viro.1998.9097

[40] Advani SJ, Brandimarti R, Weichselbaum RR, Roizman B (2000) The disappearance of cyclins A and B and the increase in activity of the G_2_/M-phase cellular kinase cdc2 in herpes simplex virus 1-infected cells require expression of the α22/U_S_1.5 and U_L_13 viral genes. J Virol 74: 8–15. 10590085 PMC111507

[41] Advani SJ, Weichselbaum RR, Roizman B (2000) The role of cdc2 in the expression of herpes simplex virus genes. Proc Natl Acad Sci USA 97: 10996–11001. 10995483 10.1073/pnas.200375297 PMC27137

[42] Poggioli GJ, Dermody TS, Tyler KL (2001) Reovirus-induced sigma 1s-dependent G(2)/M phase cell cycle arrest is associated with inhibition of p34(cdc2). J Virol 75(16): 7429–7434. 11462015 10.1128/JVI.75.16.7429-7434.2001 PMC114978

[43] Kitay MK, Rowe DT (1996) Cell cycle stage-specific phosphorylation of the Epstein-Barr virus immortalization protein EBNA-LP. J Virol 70: 7885–7893. 8892911 10.1128/jvi.70.11.7885-7893.1996 PMC190860

[44] Zafrullah M, Ozdener MH, Panda SK, Jameel S (1997) The ORF3 protein of hepatitis E virus is a phosphoprotein that associates with the cytoskeleton. J Virol 71: 9045–9053. 9371561 10.1128/jvi.71.12.9045-9053.1997 PMC230205

[45] Ye M, Duus KM, Peng J, Price DH, Grose C (1999) Varicella-zoster virus Fc receptor component gI is phosphorylated on its endodomain by a cyclin-dependent kinase. J Virol 73: 1320–1330. 9882337 10.1128/jvi.73.2.1320-1330.1999 PMC103956

[46] Fournier N, Raj K, Saudan P, Utzig S, Sahli R, Simanis V, et al (1999) Expression of human papillomavirus 16 E2 protein in Schizosaccharomyces pombe delays the initiation of mitosis. Oncogene 18: 4015–4021. 10435625 10.1038/sj.onc.1202775

[47] He J, Choe S, Walker R, Di Marzio P, Morgan DO, Landau NR (1995) Human immunodeficiency virus type 1 viral protein R (Vpr) arrests cells in the G_2_ phase of the cell cycle by inhibiting p34^cdc2^ activity. J Virol 69: 6705–6711. 7474080 10.1128/jvi.69.11.6705-6711.1995 PMC189580

[48] Goh WC, Rogel ME, Kinsey CM, Michael SF, Fultz PN, Nowak MA, et al (1998) HIV-1 vpr increases viral expression by manipulation of the cell cycle: a mechanism for selection of vpr *in vivo*. Nat Med 4: 65–71. 9427608 10.1038/nm0198-065

[49] Jang YJ, Ji JH, Choi YC, Ryu CJ, Ko SY (2007) Regulation of Polo-like kinase 1 by DNA damage in mitosis. Inhibition of mitotic PLK-1 by protein phosphatase 2A. J Biol Chem 82(4): 2473–2482. 10.1074/jbc.M605480200 17121863

[50] Davy CE, Jackson DJ, Raj K, Peh WL, Southern SA, Das P, et al (2005) Human papillomavirus type 16 E1 E4-induced G2 arrest is associated with cytoplasmic retention of active Cdk1/cyclin B1 complexes. J Virol 79(7): 3998–4011. 15767402 10.1128/JVI.79.7.3998-4011.2005 PMC1061520

[51] Bhattacharya B, Noad RJ, Roy P (2007) Interaction between Bluetongue virus outer capsid protein VP2 and vimentin is necessary for virus egress. Virol J 4: 7 17224050 10.1186/1743-422X-4-7 PMC1783847

[52] Armer H, Moffat K, Wileman T, Belsham GJ, Jackson T, Duprex WP, et al (2008) Foot-and-mouth disease virus, but not bovine enterovirus, targets the host cell cytoskeleton via the nonstructural protein 3Cpro. J Virol 82: 10556–10566. 10.1128/JVI.00907-08 18753210 PMC2573224

[53] Kanlaya R, Pattanakitsakul SN, Sinchaikul S, Chen ST, Thongboonkerd V (2010) Vimentin interacts with heterogeneous nuclear ribonucleoproteins and dengue nonstructural protein 1 and is important for viral replication and release. Mol Biosyst 6:795–806. 10.1039/b923864f 20567765

[54] Ghosh S, Ahrens WA, Phatak SU, Hwang S, Schrum LW, Bonkovsky HL (2011). Association of filamin A and vimentin with hepatitis C virus proteins in infected human hepatocytes. J Viral Hepat 18: e568–e577. 10.1111/j.1365-2893.2011.01487.x 21914078

[55] Wang X, Zhang S, Sun C, Yuan ZG, Wu X, Wang D, et al (2011) Proteomic profiles of mouse neuro N2a cells infected with variant virulence of rabies viruses. J Microbiol Biotechnol 21: 366–373. 21532319

[56] Plummer EM, Thomas D, Destito G, Shriver LP, Manchester M (2012) Interaction of cowpea mosaic virus nanoparticles with surface vimentin and inflammatory cells in atherosclerotic lesions. Nanomedicine 7: 877–888. 10.2217/nnm.11.185 22394183 PMC3567616

[57] Du N, Cong H, Tian H, Zhang H, Zhang W, Song L, et al (2014) Cell surface vimentin is an attachment receptor for enterovirus 71. J Virol 88(10): 5816–5833. 10.1128/JVI.03826-13 24623428 PMC4019121

[58] Gladue DP, O'Donnell V, Baker-Branstetter R, Holinka LG, Pacheco JM, Fernández Sainz I, et al (2013) Foot-and-mouth disease virus modulates cellular vimentin for virus survival. J Virol 87(12): 6794–803. 10.1128/JVI.00448-13 23576498 PMC3676138

[59] Zhao RY, Elder RT (2005)Viral infections and cell cycle G2/M regulation. Cell Res 15(3): 143–149. 15780175 10.1038/sj.cr.7290279

[60] Li S, Brignole C, Marcellus R, Thirlwell S, Binda O, McQuoid MJ, et al (2009) The adenovirus E4orf4 protein induces G2/M arrest and cell death by blocking protein phosphatase 2A activity regulated by the B55 subunit. J Virol 83(17): 8340–8352. 10.1128/JVI.00711-09 19535438 PMC2738146

[61] Vázquez-Iglesias L, Lostalé-Seijo I, Martínez-Costas J, Benavente J (2009) Avian reovirus sigmaA localizes to the nucleolus and enters the nucleus by a nonclassical energy- and carrier-independent pathway. J Virol 83(19): 10163–10175. 10.1128/JVI.01080-09 19640987 PMC2747991

