# Supplementary figures and images for: Suppression of Vimentin Phosphorylation by the Avian Reovirus p17 through Inhibition of CDK1 and Plk1 Impacting the G2/M Phase of the Cell Cycle
Source: PLoS One. 2016 Sep 7;11(9):e0162356. doi: 10.1371/journal.pone.0162356 (PMC5014334; doi:10.1371/journal.pone.0162356)

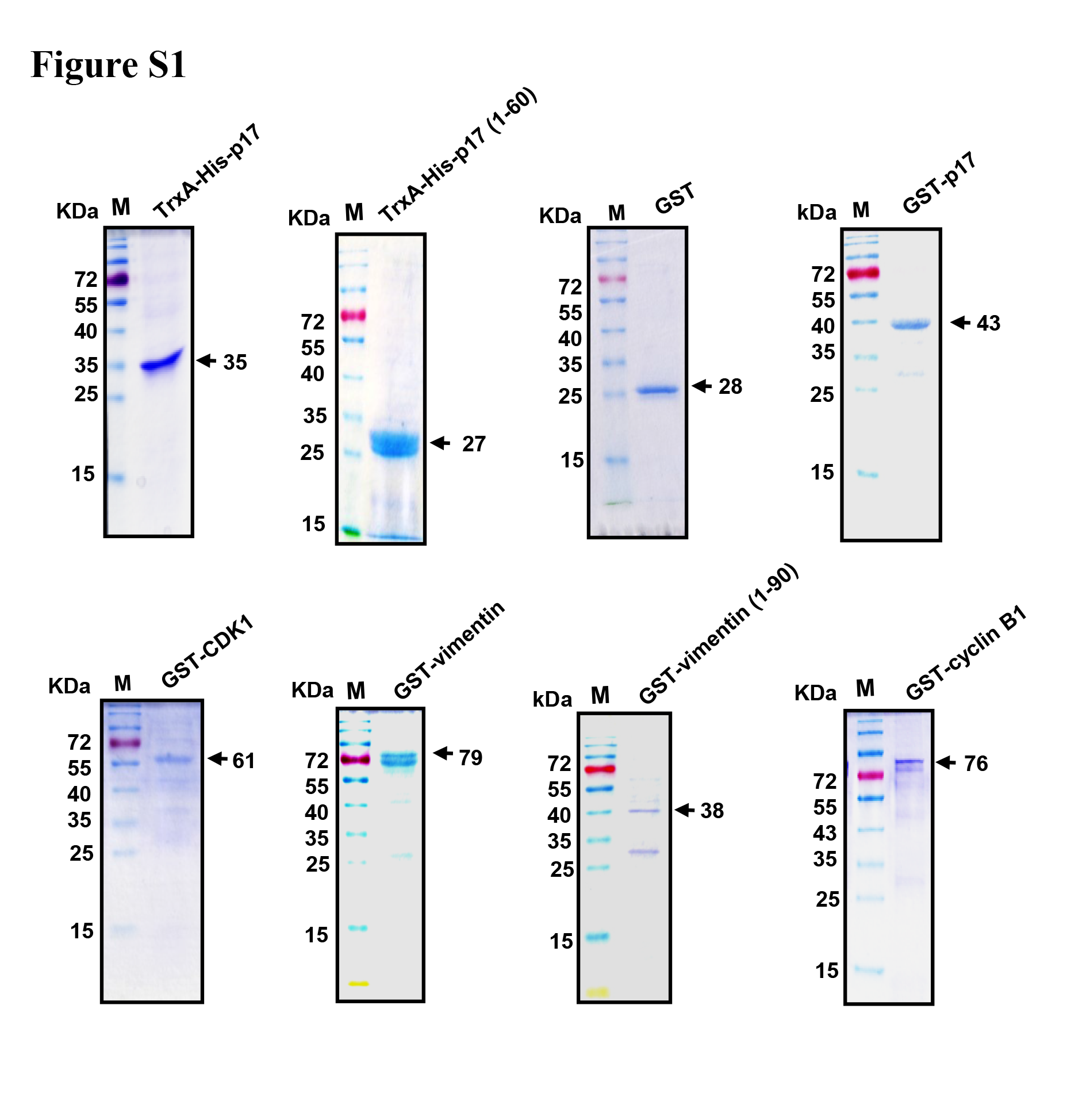

Supplement: S1 Fig — The p17 gene of ARV S1133 strain, CDK1, cyclin B1, and vimentin genes were amplified by PCR using primer pairs as indicated in Table 2. As mentioned in Material and Method section, the amplified PCR products were cut with respective restriction enzymes and then introduced into the corresponding sites in either pET32a or pGEX4T-1 vectors. The procedures for expression and purification of these proteins are described in the Material and Method section. The purified proteins were electrophoresed in 12% polyacrylamide gels at 70 V through the stacking gel and at 100 V through the resolving gels. The uncropped gels with molecular weights are shown in S9 Fig. (TIF) [file pone.0162356.s001.tif]

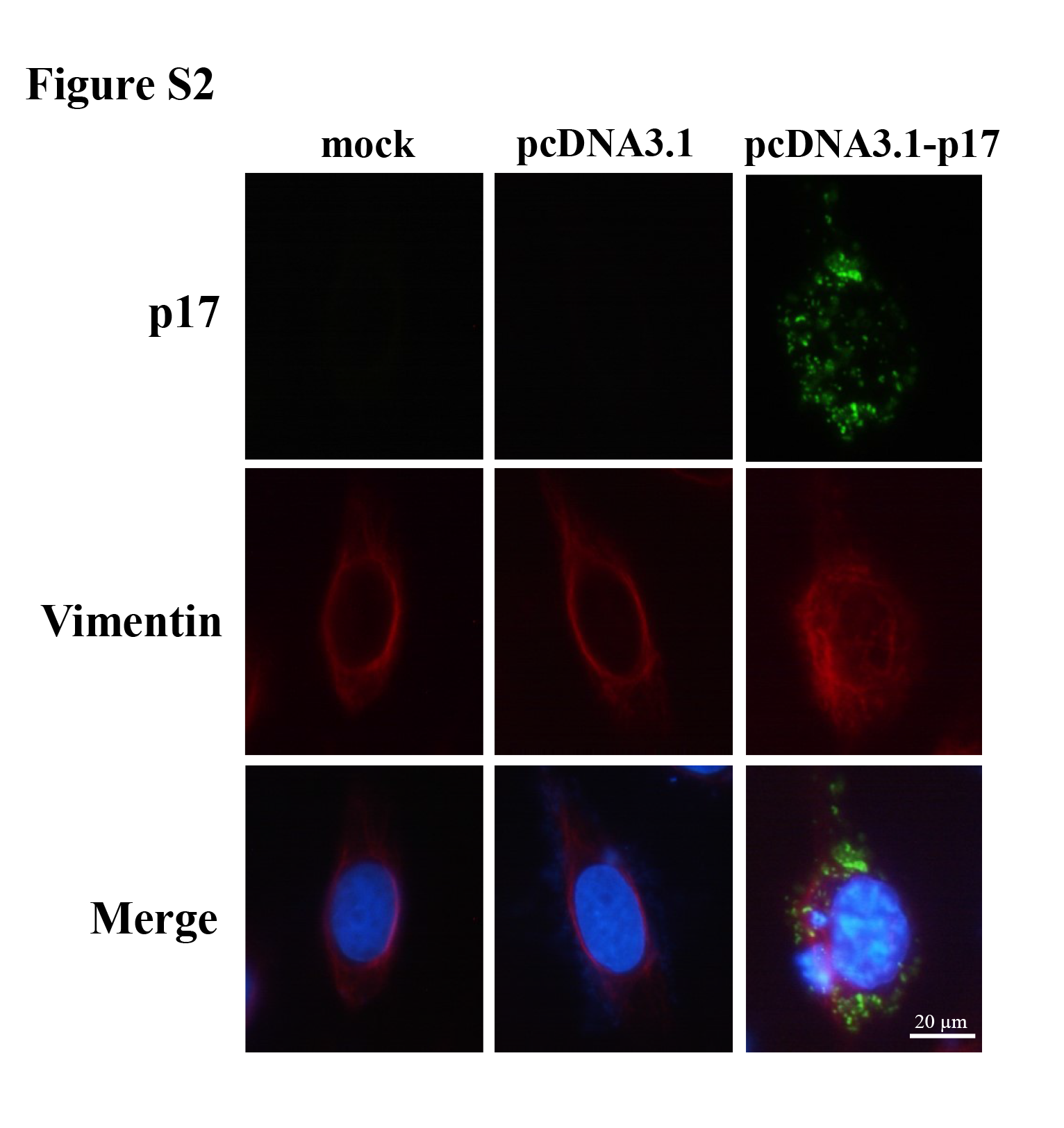

Supplement: S2 Fig — Vero cells were transfected with pcDNA3.1-p17 plasmid for 24 hours, followed by immunofluorescence staining by the indicated antibodies. Colocalization of p17 and vimentin was visualized by immunofluorescence staining. (TIF) [file pone.0162356.s002.tif]

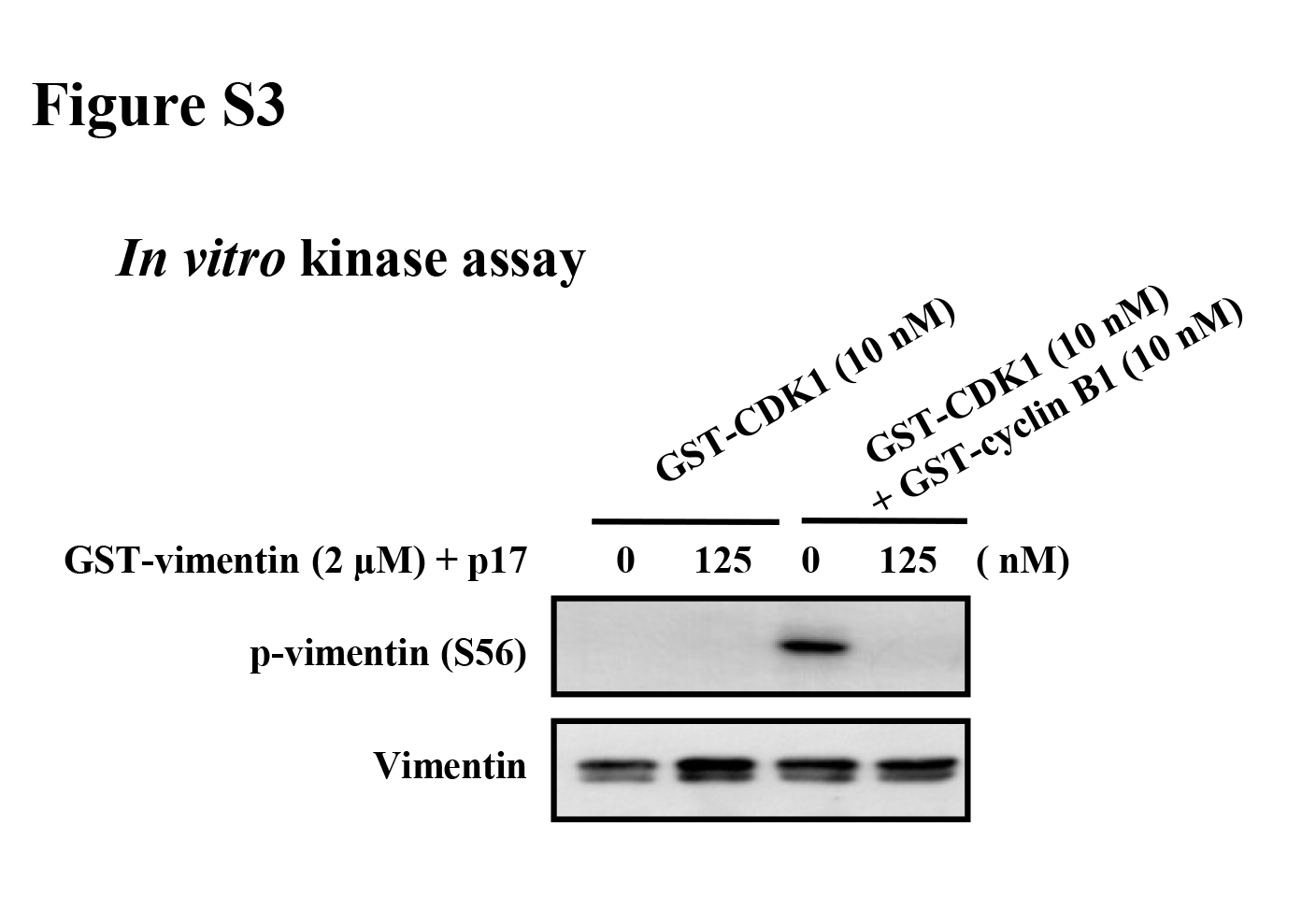

Supplement: S3 Fig — To examine whether p17 interacts with the CDK1/cyclin B1 complex leading to inhibition of CDK1 kinase activity and vimentin phosphorylation at Ser 56, an in vitro kinase assay using GST-vimentin as a substrate was performed. TrxA-His-p17 and GST-vimentin were added after 30 min incubation of GST-CDK1 and GST-cyclin B1proteins. (TIF) [file pone.0162356.s003.tif]

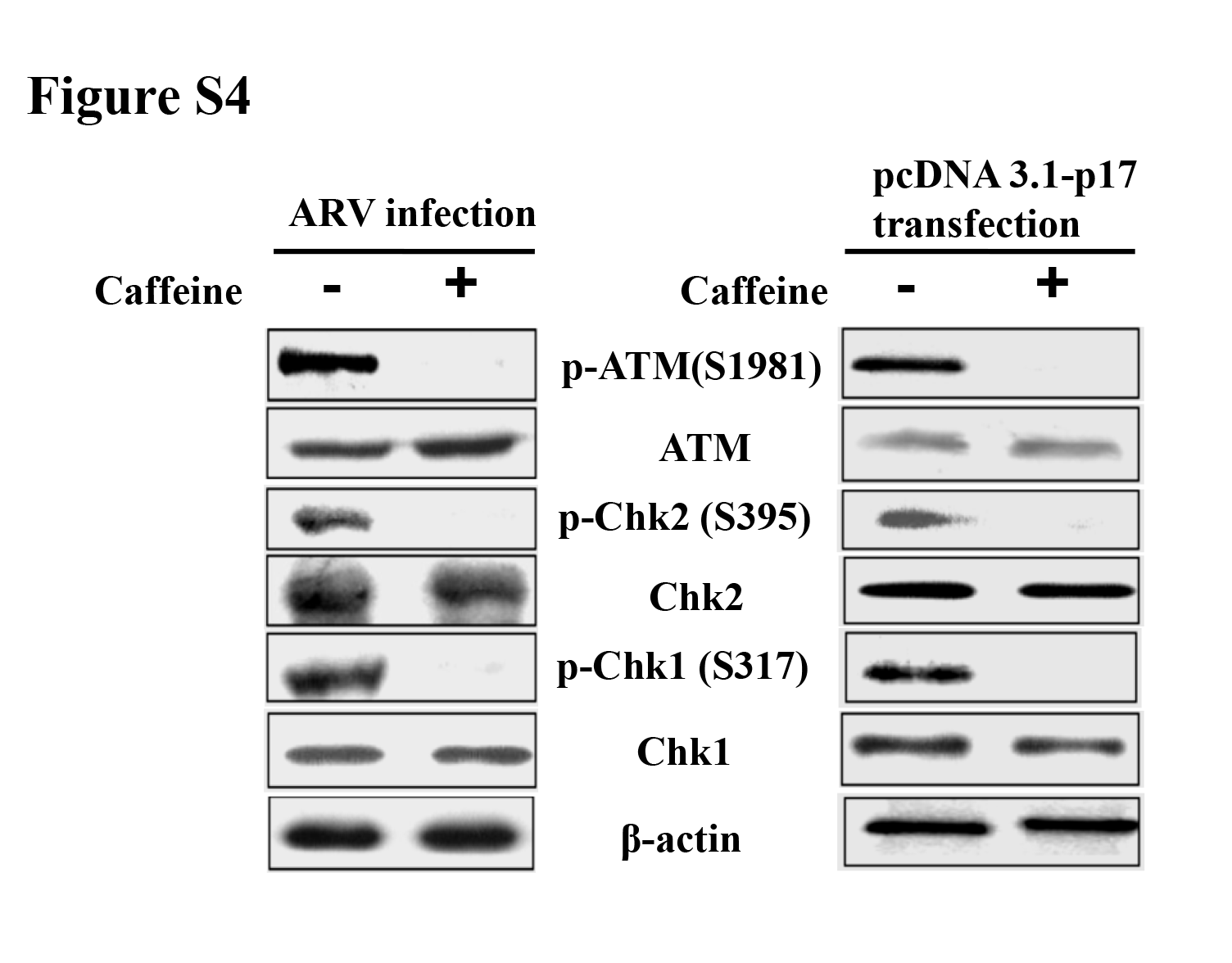

Supplement: S4 Fig — Vero cells were pretreatment with caffeine (2 mM) for 1h, followed by infection with ARV at a MOI of 10 (A) or transfection with pcDNA3.1-p17 plasmid (B) for 24 h. Cell lysates were collected and analyzed by Western blot assays with the indicated antibodies. Experiments were repeated three times, and representative blots are shown. (TIF) [file pone.0162356.s004.tif]

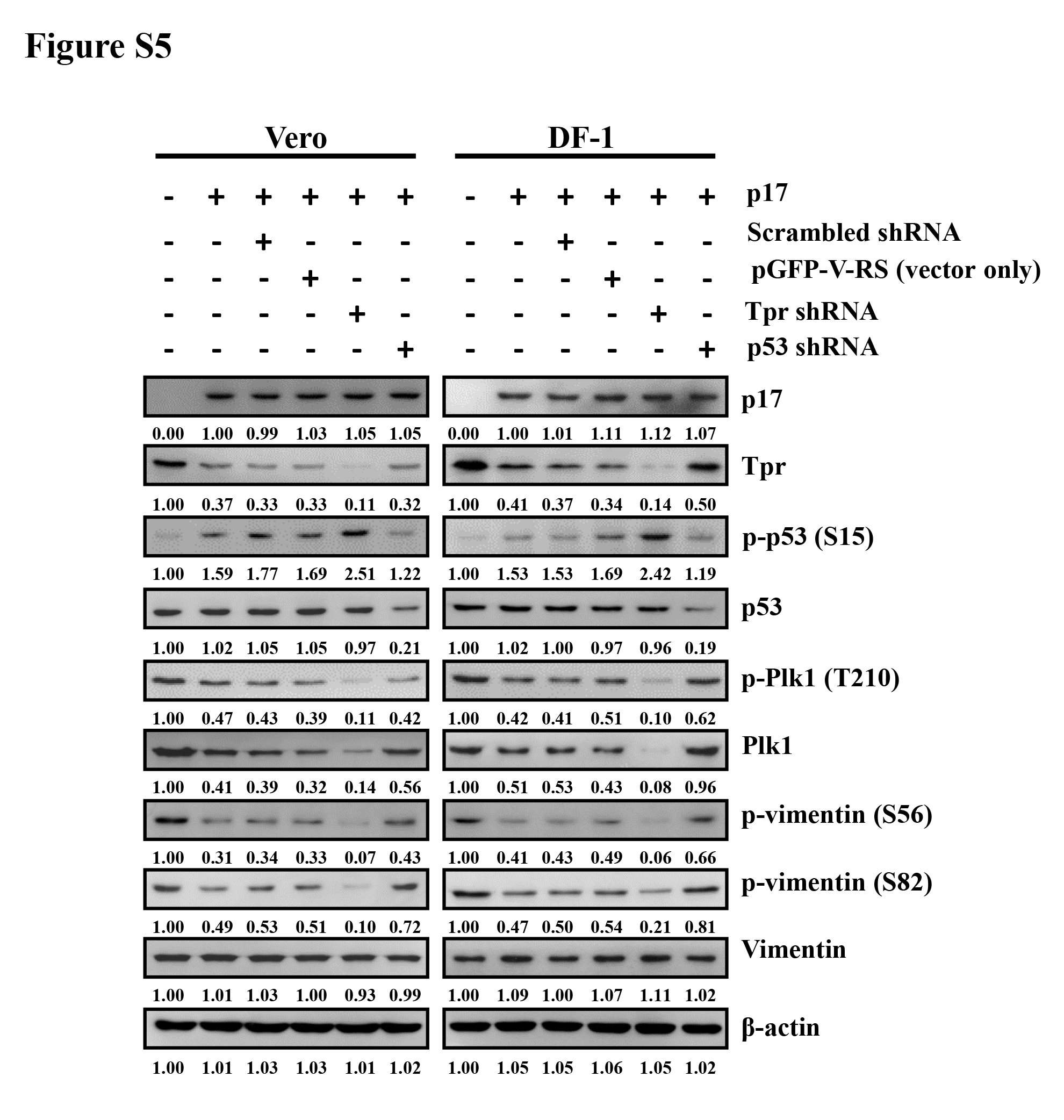

Supplement: S5 Fig — Vero (left panel) and DF-1 cells (right panel) were co-transfected with pcDNA3.1-p17, Tpr shRNA, p53 shRNA, scramble shRNA, and pGFP-V-RS (vector only), respectively, for 24 hours. The expression levels of indicated proteins were examined in p17and Tpr shRNA-co-transfected cells as well as p17 and p53 shRNA-cotransfected cells. The phosphorylated forms of p53, Plk1 and vimentin were analyzed by Western blot assays with the indicated antibodies. Cell lysates were collected and phosphorylation and protein levels were analyzed by Western blot assays. The protein levels were normalized to that for β-actin. The fold activation and inactivation indicated below each lane were normalized against the values for mock-transfection. The levels of the indicated proteins in the mock controls were considered 1-fold. The uncropped blots with molecular weights are shown in S10 Fig. (TIF) [file pone.0162356.s005.tif]

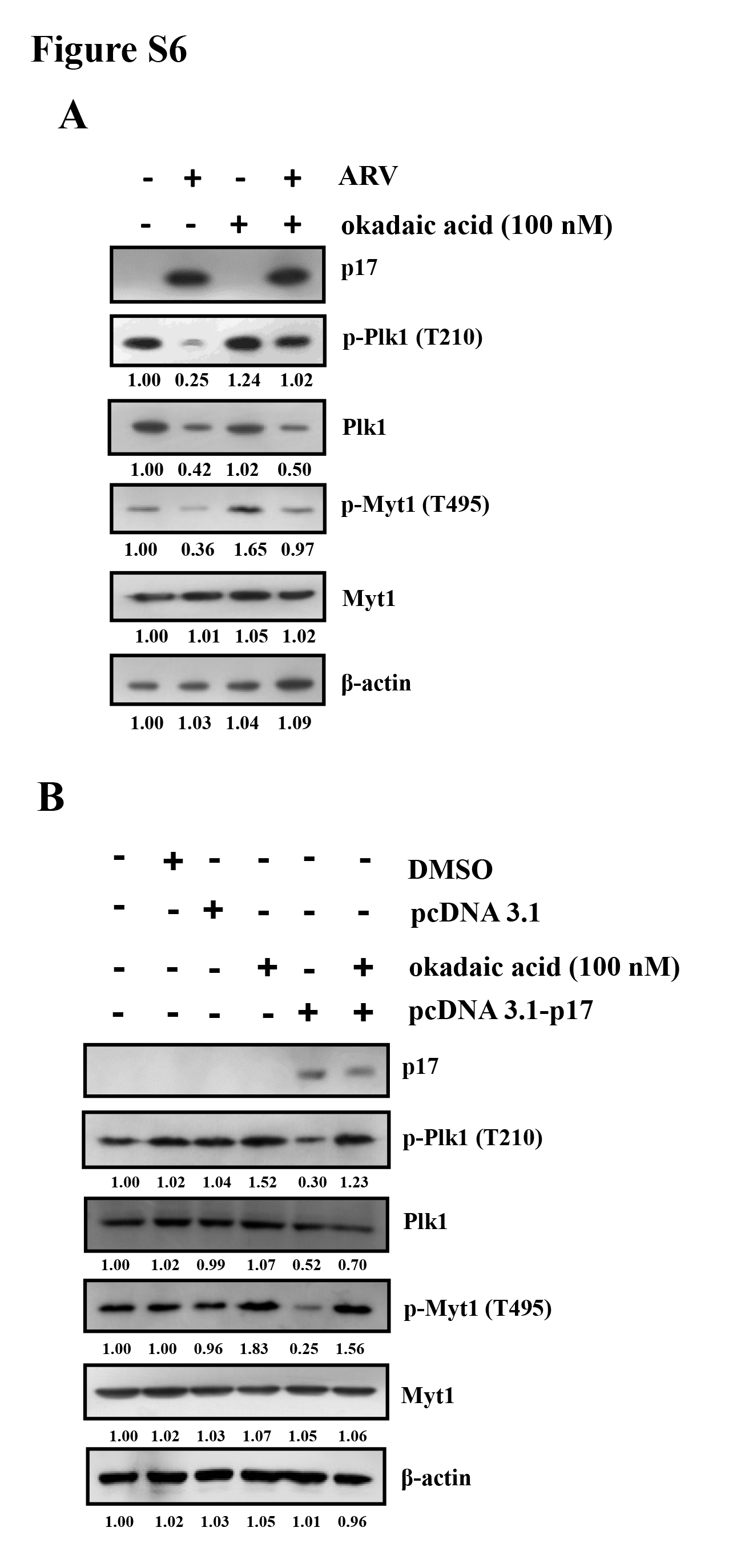

Supplement: S6 Fig — Vero cells were pretreatment with PP2A inhibitor okadaic acid (100 nM) for 1h, followed by infection with ARV at a MOI of 10 (A) or transfection with pcDNA3.1-p17 plasmid (B) for 24 h. The phosphorylated forms of p-Plk1 (T210) and p-Myt1 (T495) were analyzed by Western blot assays with the indicated antibodies. The protein levels were normalized to that for β-actin. The fold activation and inactivation indicated below each lane were normalized against the values for mock-infection or mock-transfection. The levels of the indicated proteins in the mock controls were considered 1-fold. Experiments were repeated three times, and representative blots are shown. The uncropped blots with molecular weights are shown in S10 Fig. (TIF) [file pone.0162356.s006.tif]

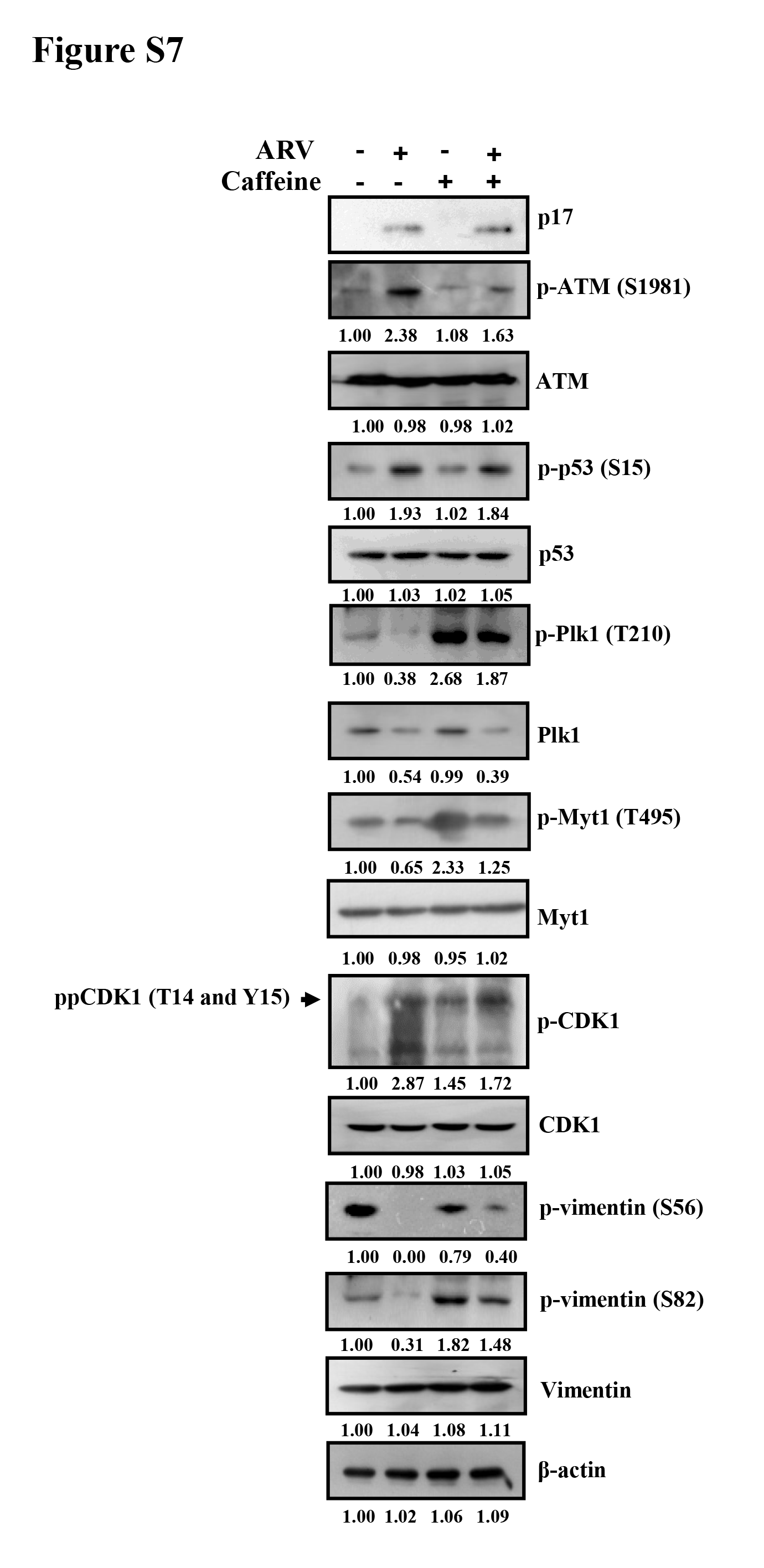

Supplement: S7 Fig — Vero cells were pretreated with caffeine (2 mM) for 1h, followed by infection with ARV at a MOI of 10 (A) or transfection with pcDNA3.1-p17 plasmid (B) for 24 h. Cell lysates were collected and analyzed by Western blot assays with the indicated antibodies. The protein levels were normalized to that for β-actin. The fold activation and inactivation indicated below each lane were normalized against the values for mock-infection. The levels of the indicated proteins in the mock controls were considered 1-fold. Experiments were repeated three times, and representative blots are shown. The uncropped blots with molecular weights are shown in S10 Fig. (TIF) [file pone.0162356.s007.tif]

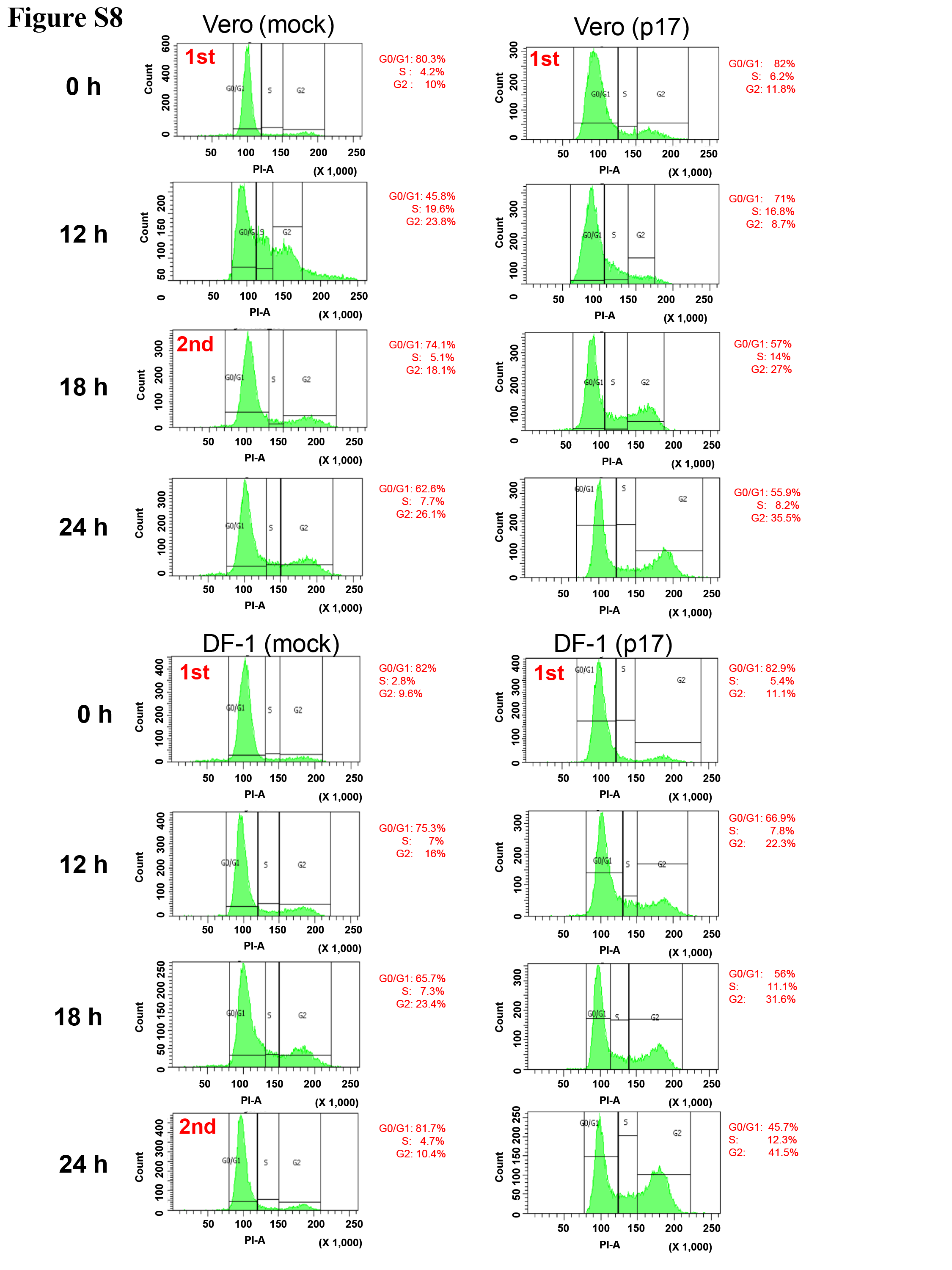

Supplement: S8 Fig — The phases in the cell cycle at which p17 inhibits cellular proliferation in both p17-transfected DF-1 and Vero cells by using flow cytometry are shown. Vero cells require 16 hours to complete a round of cell cycle while DF-1 cells need 24 hours. (TIF) [file pone.0162356.s008.tif]

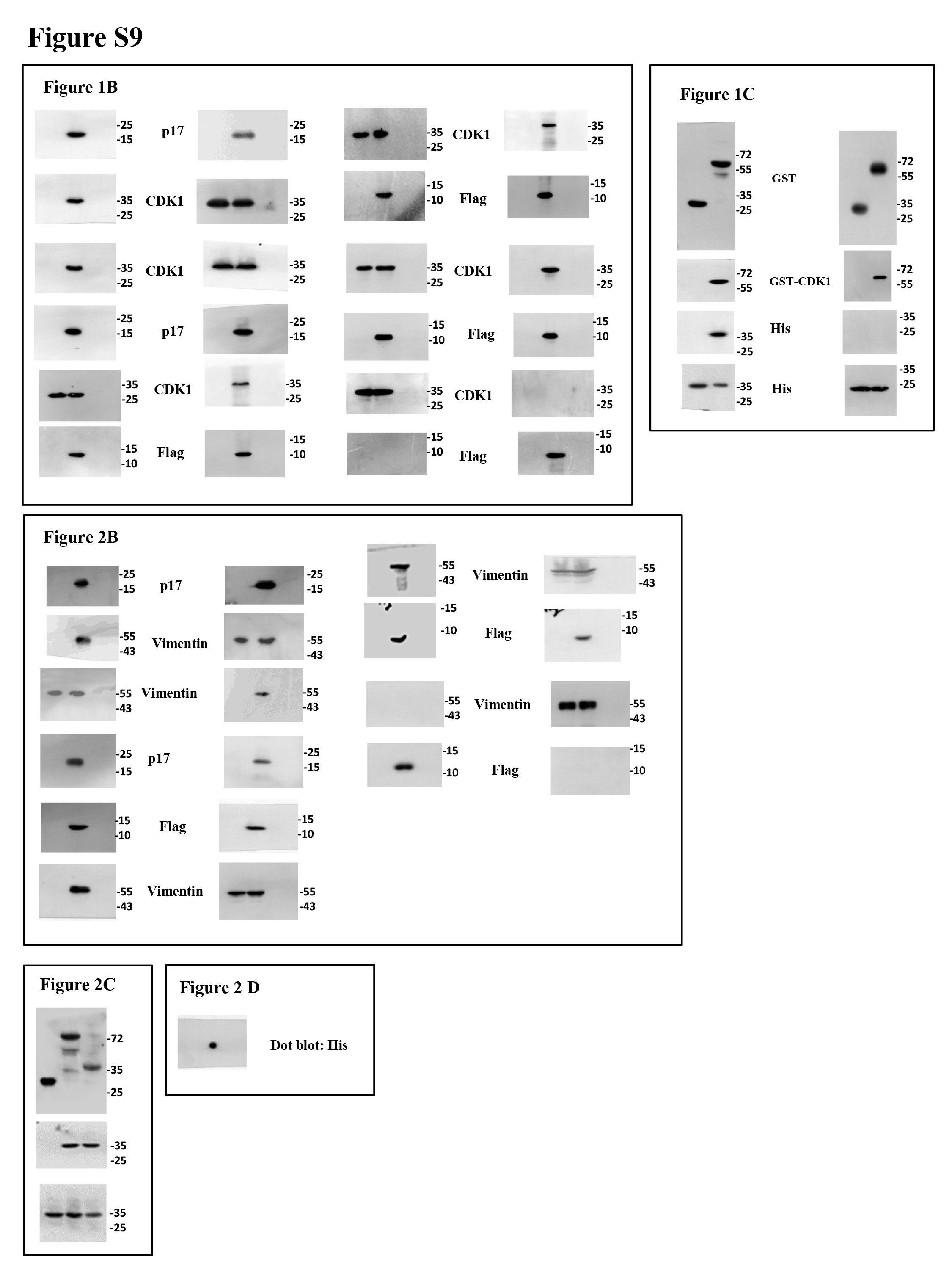

Supplement: S9 Fig — (TIF) [file pone.0162356.s009.tif]

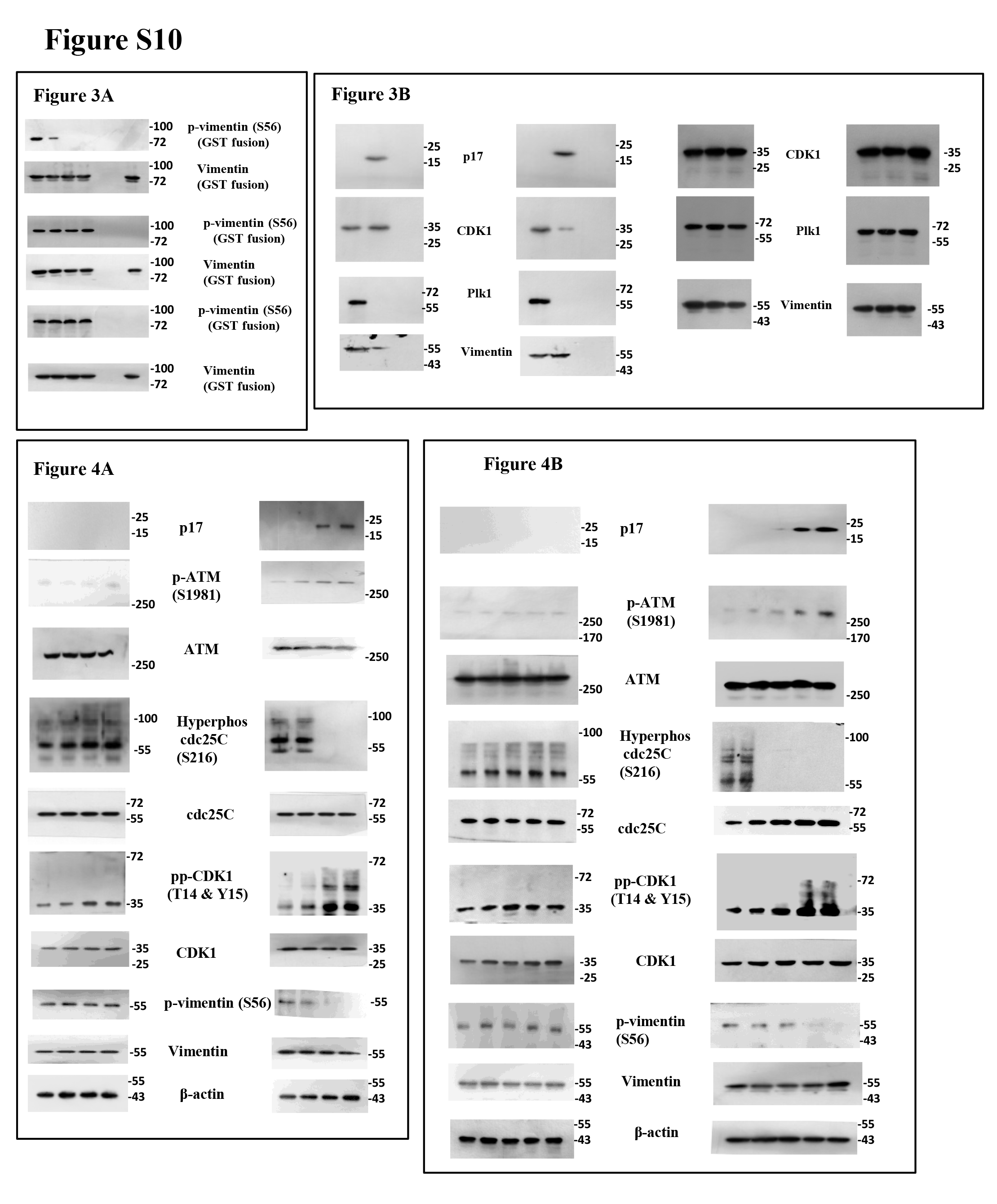

Supplement: S10 Fig — (TIF) [file pone.0162356.s010.tif]

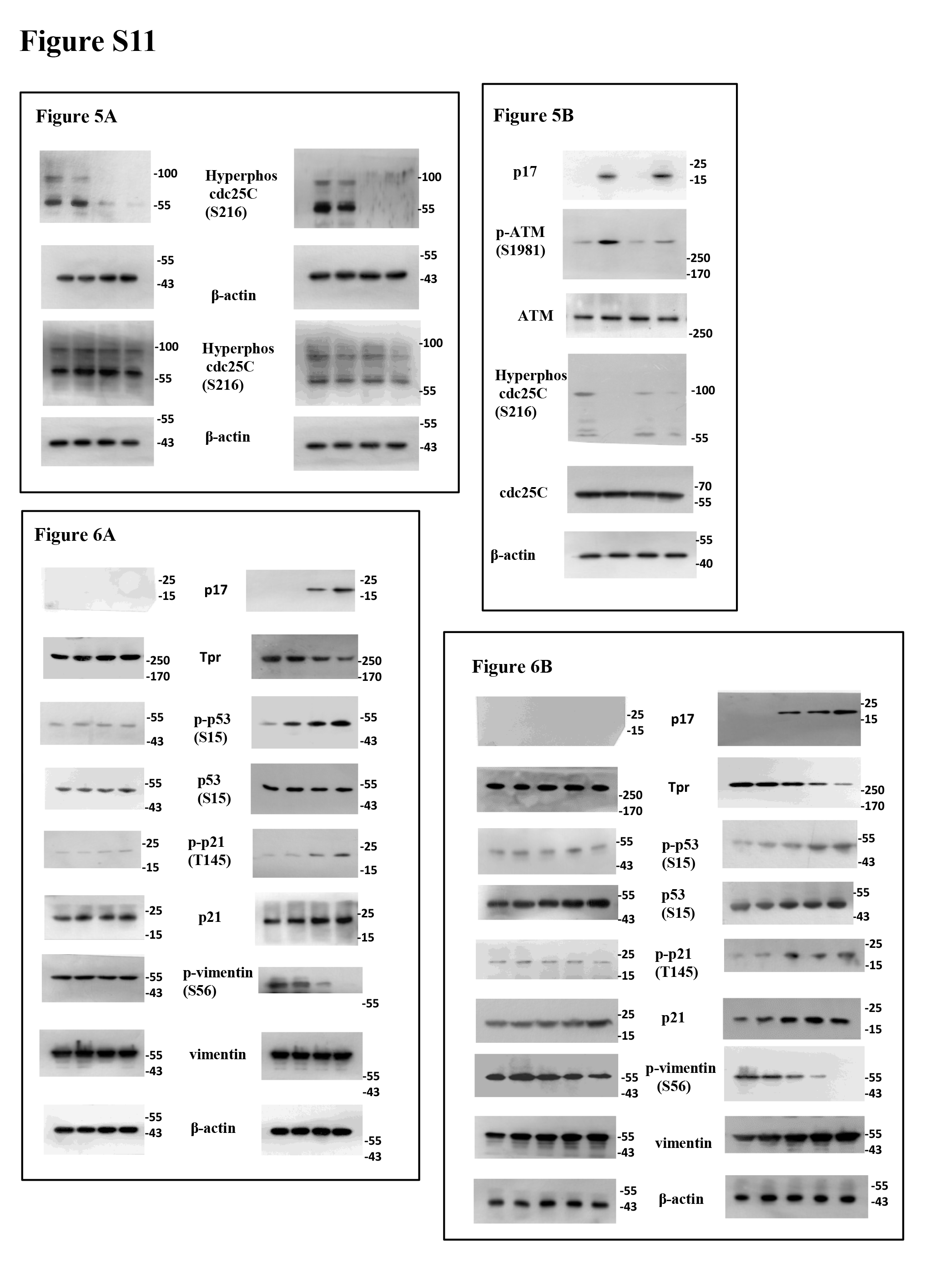

Supplement: S11 Fig — (TIF) [file pone.0162356.s011.tif]

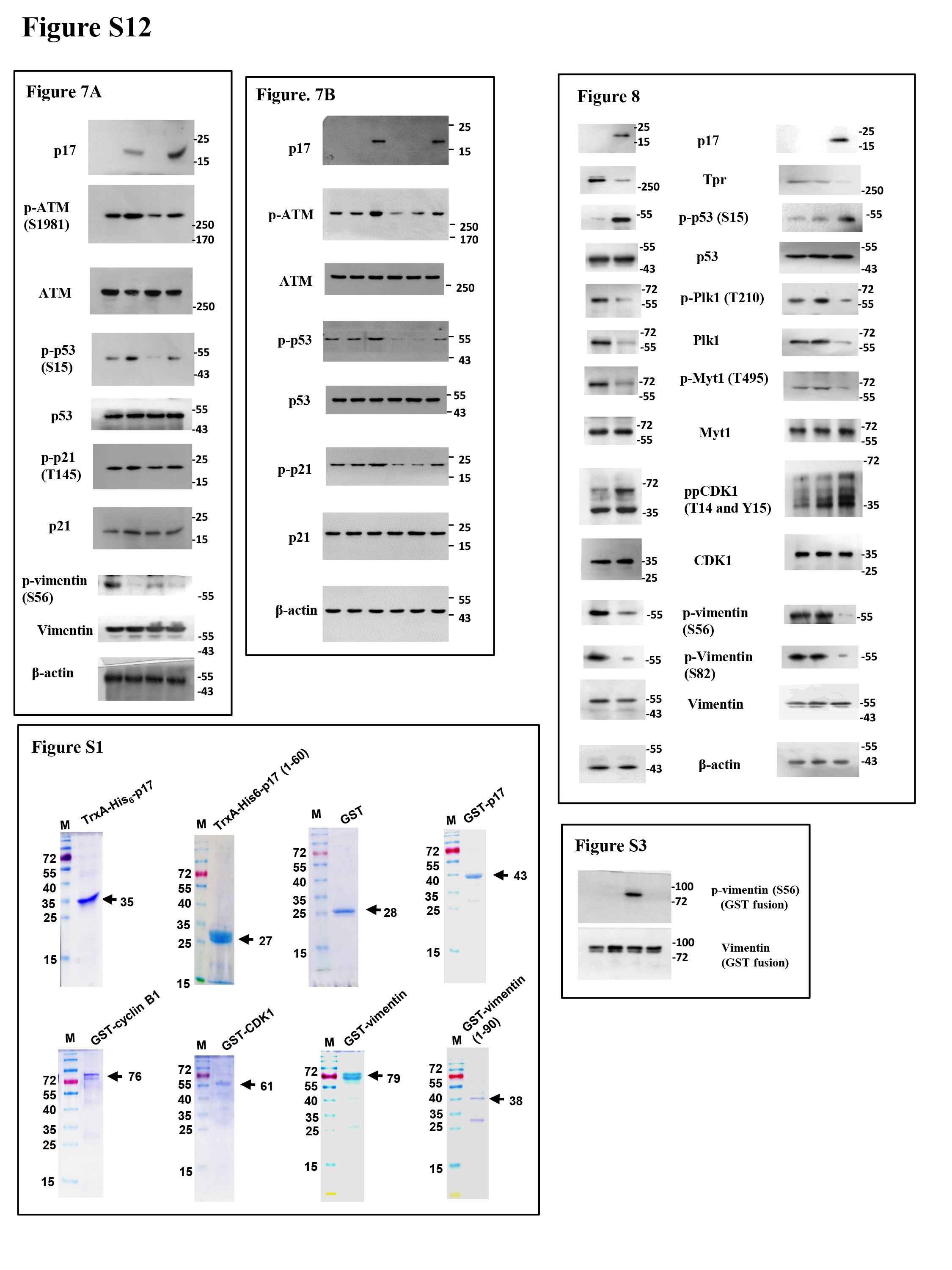

Supplement: S12 Fig — (TIF) [file pone.0162356.s012.tif]

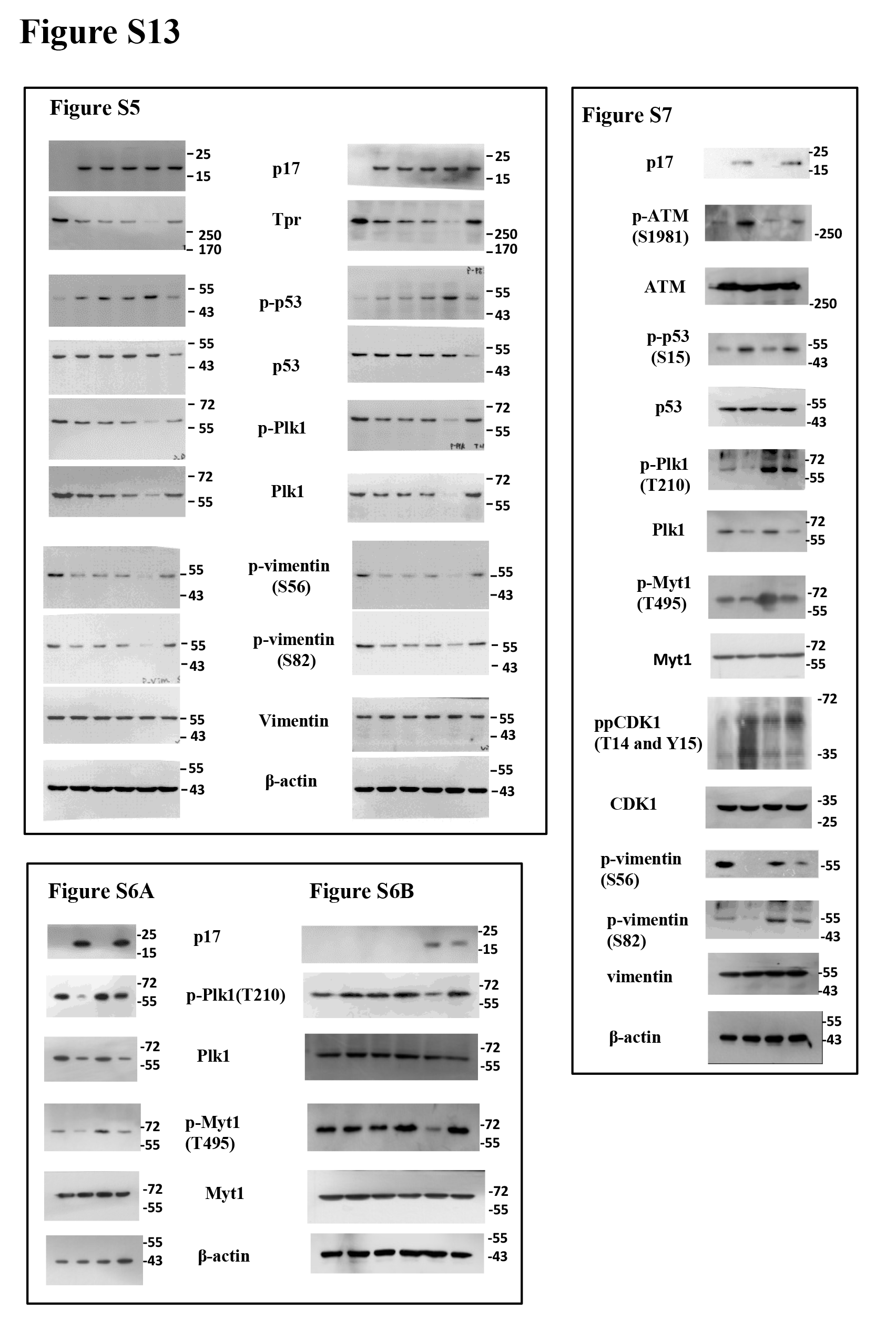

Supplement: S13 Fig — (TIF) [file pone.0162356.s013.tif]
